# Effect of Concurrent Chemoradiotherapy With Nedaplatin vs Cisplatin on the Long-term Outcomes of Survival and Toxic Effects Among Patients With Stage II to IVB Nasopharyngeal Carcinoma: A 5-Year Follow-up Secondary Analysis of a Randomized Clinical Trial

**DOI:** 10.1001/jamanetworkopen.2021.38470

**Published:** 2021-12-20

**Authors:** Qing-Nan Tang, Li-Ting Liu, Bin Qi, Shan-Shan Guo, Dong-Hua Luo, Rui Sun, Xue-Song Sun, Dong-Ping Chen, Ling Guo, Hao-Yuan Mo, Pan Wang, Sai-Lan Liu, Yu-Jing Liang, Xiao-Yun Li, Zhen-Chong Yang, Qiu-Yan Chen, Hai-Qiang Mai, Lin-Quan Tang

**Affiliations:** 1 Sun Yat-sen University Cancer Center, State Key Laboratory of Oncology in South China, Collaborative Innovation Center for Cancer Medicine, Guangdong Key Laboratory of Nasopharyngeal Carcinoma Diagnosis and Therapy, Guangzhou, China; 2 Department of Nasopharyngeal Carcinoma, Sun Yat-sen University Cancer Center, Guangzhou, China; 3 Department of Radiation Oncology, Affiliated Cancer Hospital and Institute of Guangzhou Medical University, Guangzhou, China

## Abstract

**Importance:**

Nedaplatin-based concurrent chemoradiotherapy (CCRT) regimen at 2 years was noninferior to cisplatin-based regimen in patients with locoregional, stage II to IVB nasopharyngeal carcinoma (NPC) and was associated with fewer late adverse events, but longer-term outcomes and toxicity are unclear.

**Objective:**

To evaluate the 5-year outcomes and late toxicity profile of nedaplatin-based CCRT in patients with locoregional, stage II to IVB NPC.

**Design, Settings, and Participants:**

This 5-year follow-up secondary analysis of an open-label, noninferiority, multicenter randomized clinical trial enrolled patients with nonkeratinizing stage II to IVB NPC between January 16, 2012, and July 16, 2014, with a median follow-up duration of 78 months (IQR, 3-99 months). Data analysis was conducted from November 10, 2020, to July 8, 2021.

**Interventions:**

Patients were randomly assigned (1:1) to receive nedaplatin (100 mg/m^2^)– or cisplatin (100 mg/m^2^)–based chemotherapy every 3 weeks for 3 cycles concurrently with intensity-modulated radiotherapy.

**Main Outcomes and Measures:**

The primary end point was progression-free survival (PFS). Secondary end points were overall survival, distant metastasis–free survival, and locoregional relapse–free survival.

**Results:**

A total of 402 eligible participants were enrolled (median [IQR] age, 45 [18-65] years; 302 [75.1%] male). Patients were randomly assigned to receive nedaplatin- or cisplatin-based CCRT (n = 201 for each): 196 patients (97.5%) started nedaplatin-based CCRT and 197 patients (98.0%) started cisplatin-based CCRT. Intention-to-treat analysis demonstrated a 5-year progression-free survival rate of 81.4% (95% CI, 75.9%-86.9%) for the cisplatin group and 79.8% (95% CI, 74.1%-85.5%) for nedaplatin group, with a difference of 1.6% (95% CI, −6.3% to 9.5%; *P* = .002 for noninferiority). No significant survival differences were observed between the cisplatin and nedaplatin groups for 5-year overall survival (89.4% vs 88.8%, *P* = .63), distant metastasis–free survival (85.9% vs 90.4%, *P* = .17), and locoregional relapse–free survival (92.6% vs 89.6%, *P* = .17) rates. The cisplatin group had a higher incidence of grade 3 and 4 auditory toxic effects than the nedaplatin group (35 [17.7%] vs 21 [10.5%], *P* = .04).

**Conclusions and Relevance:**

In this secondary analysis of a randomized clinical trial, long-term analysis confirmed that nedaplatin-based CCRT could be regarded as an alternative doublet treatment strategy to cisplatin-based CCRT in stage II to IVB NPC.

**Trial Registration:**

ClinicalTrials.gov Identifier: NCT01540136

## Key Points

**Question:**

Does nedaplatin-based concurrent chemoradiotherapy (CCRT) have comparable efficacy to cisplatin-based CCRT in stage II to IVB nasopharyngeal carcinoma?

**Findings:**

In this 5-year follow-up secondary analysis of a randomized clinical trial of 402 eligible patients, nedaplatin-based CCRT achieved 5-year progression-free survival rates comparable to those in the cisplatin-based CCRT group. Patients in the nedaplatin group also experienced fewer late toxic effects.

**Meaning:**

These results confirm that nedaplatin-based CCRT can be regarded as an alternative to cisplatin-based CCRT in stage II to IVB nasopharyngeal carcinoma.

## Teaser

This 5-year follow-up secondary analysis of a randomized clinical trial analyzed survival outcomes and late toxic effects to assess the efficacy of nedaplatin-based concurrent chemoradiotherapy in the treatment of stage II to IVB nasopharyngeal carcinoma.

## Introduction

Nasopharyngeal carcinoma (NPC) is endemic in Southeast Asia and Southern China, and an age-standardized incidence rate of 3.0 to 10.2 per 100 000 population has been reported in China.^1,2,3^ On the basis of its epidemiologic outcomes, aggressive behavior, and coexistence with Epstein-Barr virus (EBV) infection, NPC can be differentiated from other head and neck malignant tumors.^4,5^ Cisplatin-based concurrent chemoradiotherapy (CCRT) has been established as the mainstay and standard of care for locoregionally advanced NPC based on several meta-analyses and prospective randomized clinical trials.^6,7,8^ Radiotherapy administered concurrently with 100 mg/m^2^ of cisplatin every 3 weeks is recommended by the National Comprehensive Cancer Network for patients with stage II to IVB NPC. However, the addition of cisplatin-based chemotherapy to radiotherapy increases the frequency of treatment-related toxic effects, including severe gastrointestinal responses, hearing deficits, renal toxic effects, and neurotoxic effects, which decrease treatment adherence and the quality of life of patients.^6^ Therefore, another antitumor drug with similar therapeutic efficacy and fewer adverse effects is urgently needed.

Nedaplatin, a cisplatin analogue, has antitumor mechanisms and effectiveness similar to cisplatin and was designed to decrease the adverse events, such as nephrotoxic and gastrointestinal toxic effects, seen with cisplatin. Nedaplatin is an effective and well-tolerated chemotherapeutic agent in various malignant tumors.^9,10,11^ In vitro, nedaplatin is a potentially radiosensitizing agent in NPC and cervical squamous cell carcinoma cells.^12^ A retrospective study^13^ and 2 phase 2 trials^14,15^ found that neoadjuvant chemotherapy with nedaplatin plus fluorouracil or docetaxel followed by nedaplatin-based CCRT is an effective treatment with relatively good adherence from patients with stage II to IVB NPC. Thus, nedaplatin represents a potential alternative drug to cisplatin.

To obtain a comprehensive understanding of the comparative efficacy and safety of CCRT regimens composed of cisplatin and nedaplatin, we performed a phase 3 randomized clinical trial in patients with stage II to IVB NPC to investigate whether the nedaplatin-based CCRT regimen was noninferior to the cisplatin-based regimen. The initial 2-year results indicated that nedaplatin-based CCRT is noninferior to cisplatin-based CCRT, with a difference of 1.9% (95% CI, −4.2 to 8.0; *P* = .005 for noninferiority) for progression-free survival (PFS) in the intention-to-treat population and 1.0% (95% CI, −5.2 to 7.0; *P* = .002 for noninferiority) in the per-protocol population, although there was limited information on long-term follow-up and late toxic effects. We aimed to bridge the knowledge gap with a 5-year detailed analyses of survival outcomes and late toxic effects to assess the ultimate therapeutic efficacy of nedaplatin-based CCRT in stage II to IVB NPC.

## Methods

### Patient Selection

**Figure 1.**
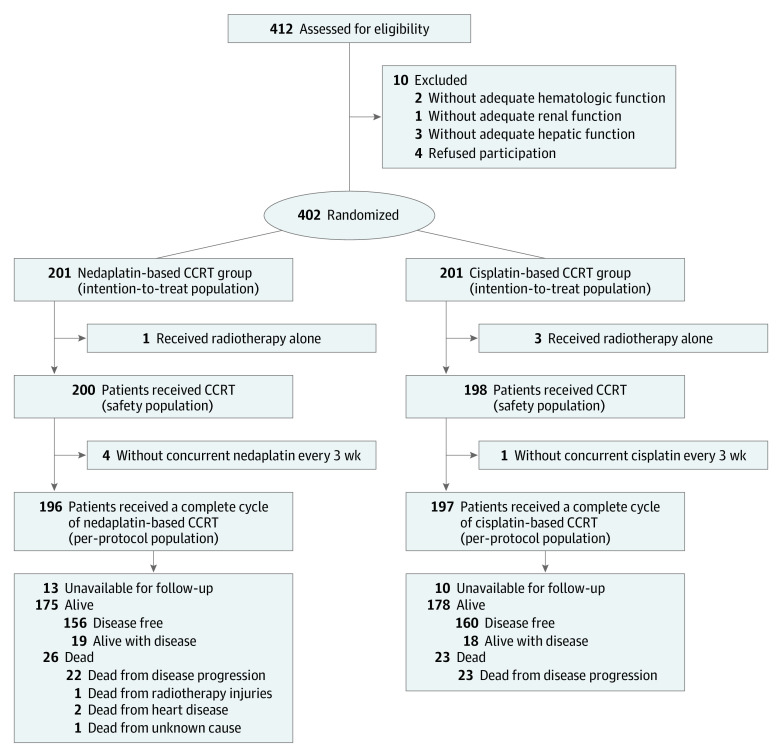
Flowchart of Patients Included and Excluded in This Study CCRT indicates concurrent chemoradiotherapy.

This 5-year follow-up secondary analysis of an open-label, noninferiority, multicenter randomized clinical trial enrolled patients with locoregionally advanced, biopsy-confirmed, newly diagnosed, nonkeratinizing NPC (type II/III World Health Organization classification). The eligibility criteria were as follows: (1) 18 to 65 years of age; (2) stage T1-4N1-3 or T3-4N0 disease (according to the 7th edition of the International Union Against Cancer/American Joint Committee on Cancer staging system); (3) no distant metastasis; (4) normal hematologic, kidney, and liver findings; and (5) Karnofsky Performance Status Scale score of 70 or higher. Patients with prior or synchronous malignant disease, previous treatment for NPC, primary distant metastasis, or pregnancy and lactating mothers were excluded. Randomization was performed by a computer-generated random number code. Randomization was stratified by treatment center and disease stage (II, III, or IV). The procedures of randomization and allocation concealment were performed according to practical guidance described previously.^16^ Eligible patients were randomly assigned (1:1 ratio) to receive nedaplatin-based or cisplatin-based CCRT with a block size of 6 (Figure 1). This study is registered in ClinicalTrials.gov and was approved (along with follow-up analyses) by the clinical research ethics committee or institutional review board at each participating center, and all patients provided written informed consent for study participation. All data were deidentified. This study followed the Consolidated Standards of Reporting Trials (CONSORT) reporting guideline.^17^ The trial protocol can be found in Supplement 1.

### Treatment and Follow-up

From January 16, 2012, to July 16, 2014, a total of 402 eligible patients from 2 institutions (Sun Yat-sen University Cancer Center and Affiliated Cancer Hospital and Institute of Guangzhou Medical University) were randomly assigned to receive nedaplatin- or cisplatin-based CCRT (n = 201 for each). All patients received intensity-modulated radiotherapy (IMRT), and the recommended dose in each fraction was 2.00 to 2.33 Gy. The accumulated dose of the planned target volumes was 66 to 70 Gy for gross tumor volume of nasopharynx, 64 to 70 Gy for gross tumor volume of lymph nodes, 60 to 62 Gy for clinical tumor volume, and 54 to 56 Gy for secondary tumor volume. We divided the accumulated dose into 30 to 33 fractions, administered daily (Monday through Friday) for 6 to 7 weeks. Patients in the nedaplatin group received concurrent nedaplatin (100 mg/m^2^; 2-hour intravenous infusion) and those in the cisplatin group received concurrent cisplatin (100 mg/m^2^; 4-hour intravenous infusion) every 3 weeks for 3 cycles.

The primary end point was PFS, calculated from the date of random assignment to the date of first relapse at any site, death from any cause, or patient censoring at last follow-up, and the secondary end points were overall survival (OS; interval from date of random assignment to the date of death from any cause or patient censoring at the last follow-up), distant metastasis–free survival (DMFS), and locoregional relapse–free survival (LRFS). Both DMFS and LRFS were determined from the date of random assignment to the date of distant relapse or locoregional relapse, respectively, death from any cause, or patient censoring at the last follow-up. During the first 3 years after treatment completion, patients were followed up for at least every 3 months and every 6 months thereafter until death. After a median follow-up time of 47 months, we compared the cisplatin and nedaplatin groups for efficacy and safety.^17^ In subsequent follow-ups, patients visited the outpatient clinic, and we recorded their survival situations and long-term toxic effects. Patients who did not return for follow-up were contacted via telephone or email to ascertain their survival status and long-term toxic effects. Magnetic resonance imaging of head and neck, fiberoptic nasopharyngoscopy, chest radiography, and abdominal sonography were performed every 6 months for the first 3 years and then every 12 months or on clinical indication of tumor recurrence. Late toxic effects were defined as those that occurred 6 months after completion of radiotherapy, which were assessed and graded based on the Radiation Therapy Oncology Group/European Organisation for Research and Treatment of Cancer morbidity-scoring schema.

### Statistical Analysis

All efficacy analyses were performed in the intention-to-treat and the per-protocol populations. Safety analyses were performed in all randomly assigned participants, excluding those who did not receive chemotherapy. The χ^2^ test or Fisher exact test was performed for categorical variables, and the Mann-Whitney *U* test was used for continuous variables to identify intergroup differences. We assessed the between-group differences in PFS and OS with the Kaplan-Meier method and obtained 95% CIs using the Greenwood formula. We used the Nelson-Aalen cumulative risk curve to access the distributions of time to locoregional relapse and distant metastasis events and performed analyses for cumulative incidence of competing risk. The Fine and Gray method was used to test the homogeneity of cumulative incidence function for distant metastasis and locoregional relapse with adjustments to explain death not related to treatment failure. Patients lost to follow-up or those with no event observed at the last contact were censored. Hazard ratios (HRs) with 95% CIs were calculated using a Cox proportional hazards regression model. Multivariate analyses using a Cox proportional hazards regression model were used to test the independent significance of potential prognostic factors. Interaction and stratified analyses were conducted on the basis of sex, age, Karnofsky Performance Status score, and cancer stage. Interaction is the situation whereby the association of one risk factor with a certain outcome variable differs across strata of another risk factor. To calculate these measures, a Cox proportional hazards regression model should be built. In our study, treatment methods and other potential prognostic factors (sex [male or female] and age [45 or ≥45 years], Karnofsky Performance Status score [70-80 or 90-100 years], and cancer stage [II-III or IVA-B]) were entered into the multivariate Cox proportional hazards regression model to test for their main effects, and an interaction term between treatment methods and the potential prognostic factors was then added into the model to test their interaction effect for survival. SPSS software, version 24.0 (SPSS Inc); Stata software, version 15.0 (StataCorp LLC); and R, version 3.5.0 (R Foundation for Statistical Computing) were used for statistical analyses. A 2-sided *P* < .05 was considered statistically significant.

## Results

### Patient Characteristics

A total of 402 eligible participants were enrolled (median [IQR] age, 45 [18-65] years; 302 [75.1%] male). Patients were randomly assigned to receive nedaplatin- or cisplatin-based CCRT (n = 201 for each): 196 patients (97.5%) started nedaplatin-based CCRT and 197 patients (98.0%) started cisplatin-based CCRT. The 2 treatment groups were well matched on baseline demographic and clinical characteristics (Table 1). Because of the inability of 1 participating institution to perform the plasma EBV-DNA test, only 174 patients (86.6%) in the nedaplatin group and 171 patients (85.1%) in the cisplatin group underwent plasma EBV-DNA measurement, and the median concentrations of plasma EBV-DNA were 2210 copies/mL (IQR, 0-19 150 copies/mL) in the nedaplatin group and 465 copies/mL (IQR, 0-11 300 copies/mL) in the cisplatin group. All surviving participants were followed up for more than 5 years (median follow-up duration for all patients, 78 months; IQR, 3-99 months).

**Table 1.** Baseline Characteristics of the Study Participants^a^

Characteristic	Nedaplatin group (n = 201)	Cisplatin group (n = 201)
Sex		
Male	144 (71.6)	158 (78.6)
Female	57 (28.3)	43 (21.4)
Age, median (range), y	44 (18-65)	45 (20-64)
Karnofsky Performance Status score		
90-100	192 (95.5)	190 (94.5)
70-80	9 (4.5)	11 (5.5)
WHO histologic grade		
II	3 (1.4)	4 (1.9)
III	198 (98.5)	197 (98.1)
T category		
T1	5 (2.5)	3 (1.4)
T2	40 (19.9)	48 (23.9)
T3	125 (62.2)	120 (59.7)
T4	31 (15.4)	30 (14.9)
N category		
N0	19 (9.5)	19 (9.5)
N1	91 (45.3)	89 (44.3)
N2	78 (38.8)	79 (39.3)
N3	13 (6.5)	14 (7.0)
Stage		
II	24 (11.9)	24 (11.9)
III	135 (67.2)	135 (67.2)
IVA	31 (15.4)	29 (14.4)
IVB	11 (5.5)	13 (6.5)
Pretreatment EBV-DNA test DNA level, copies per mL^b^		
<1500	81 (46.6)	96 (56.1)
≥1500	93 (53.4)	75 (43.9)
Median (IQR)	2210 (0-19 150)	465 (0-11 300)

Abbreviations: EBV-DNA, Epstein-Barr virus DNA; WHO, World Health Organization.

^a^ Data are presented as number (percentage) of patients unless otherwise indicated.

^b^ The plasma EBV-DNA test was optional in this trial and was not performed for all enrolled patients.

### Treatment Delivery

Overall, 196 of 201 patients (97.5%) in the nedaplatin group started nedaplatin-based CCRT (per-protocol population), 115 of 201 patients (57.2%) completed 3 cycles, and 81 of 201 patients (40.3%) discontinued (2 cycles: n = 78; 1 cycle: n = 3). In the cisplatin group, 197 of 201 patients (98.0%) started cisplatin-based CCRT (per-protocol population), 131 of 201 patients (65.2%) received all 3 cycles, and 66 of 201 patients (32.8%) received 2 cycles of concurrent cisplatin. The proportion of patients who received 3 cycles of nedaplatin-based concurrent chemotherapy was lower than patients in the cisplatin group because approximately 17% of patients in the nedaplatin group had grade 2 to 4 thrombocytopenia and the recovery time required for these events exceeded the predefined time frame of nedaplatin administration after radiotherapy completion, and these patients did not receive the third cycle of nedaplatin.^17^ The median dose per fraction was 2.33 Gy (IQR, 2.19-2.33 Gy), and the median radiotherapy dose was 70 Gy (IQR, 70-70 Gy). No significant intergroup difference was found in treatment intensity and adherence.^17^

### Efficacy

In total, of the 402 patients, 86 (21.4%) experienced disease progression, 49 (12.2%) died, 52 (12.9%) had distant metastasis, 40 (10.0%) had locoregional relapse, and 10 (2.5%) had both distant metastasis and locoregional relapse. Details of the rates of death, disease progression, distant failure, and locoregional relapse at the last follow-up are presented in eTable 1 in Supplement 2.

**Figure 2.**
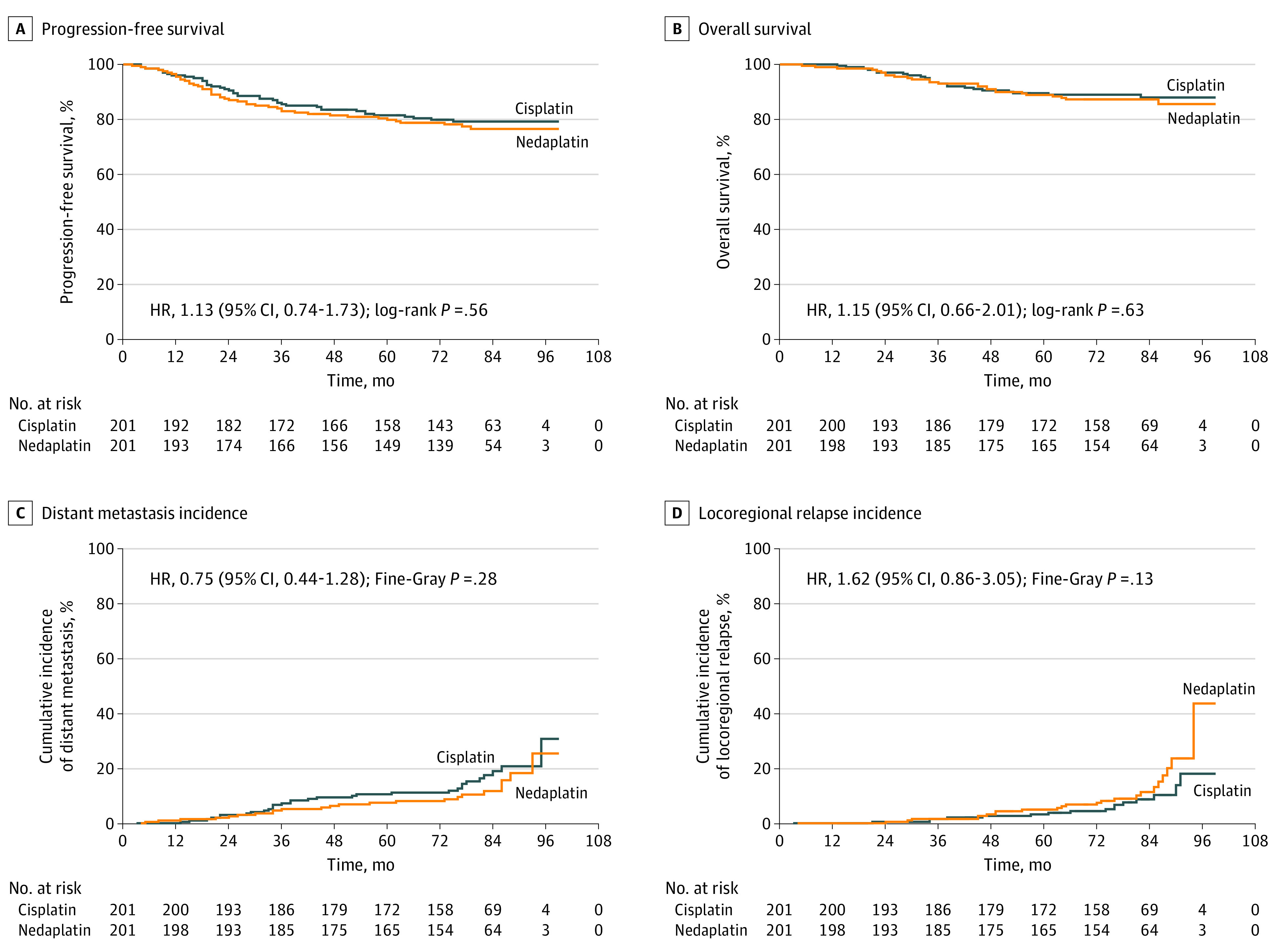
Progression-Free and Overall Survival and Cumulative Incidence of Distant Metastasis and Locoregional Relapse in the Intention-to-Treat Population HR indicates hazard ratio.

On the basis of the intention-to-treat analysis, the 5-year PFS rate was 81.4% (95% CI, 75.9%-86.9%) in the cisplatin group and 79.8% (95% CI, 74.1%-85.5%) in the nedaplatin group (HR, 1.13; 95% CI, 0.74–1.73; log-rank *P* = .56) (eTable 2 in Supplement 2; Figure 2), with a difference of 1.6% (95% CI, −6.3% to 9.5%; *P* = .002 for noninferiority), which was lower than the prespecified noninferiority margin of 10%. In the per-protocol analysis, the 5-year PFS rate was 81.1% (95% CI, 75.6%-86.6%) in the cisplatin group and 80.3% (95% CI, 74.6%-86.0%) in the nedaplatin group (HR, 1.11; 95% CI, 0.72-1.70; log-rank *P* = .64) (eTable 2 and eFigure 1 in Supplement 2), with a difference of 0.8% (95% CI, −7.1 to 8.7; *P* = .003 for noninferiority). With regard to OS, no statistically significant difference was found between the cisplatin and nedaplatin groups in both the intention-to-treat (89.4% vs 88.8%; HR, 1.15; 95% CI, 0.66-2.01; log-rank *P* = .63) (eTable 2 in Supplement 2; Figure 2) and per-protocol (89.2% vs 89.0%; HR, 1.11; 95% CI, 0.63-1.95; log-rank *P* = .72) (eTable 2 and eFigure 1 in Supplement 2) populations.

The cumulative incidence of distant metastasis (26.9% vs 22.8%; HR, 0.75; 95% CI, 0.44-1.28; Fine-Gray *P* = .28) (Figure 2) and the cumulative incidence of locoregional relapse (16.7% vs 21.2%; HR, 1.62; 95% CI, 0.86-3.05; Fine-Gray *P* = .13) (Figure 2) did not significantly differ between the cisplatin and nedaplatin groups in the intention-to-treat analysis, and similar results were observed in the per-protocol analysis (eFigure 1 in Supplement 2). The 2 treatment arms did not differ regarding PFS rates, OS rates, the cumulative incidence of distant metastasis rates, and locoregional relapse rates.

### Subgroup Analyses

**Figure 3.**
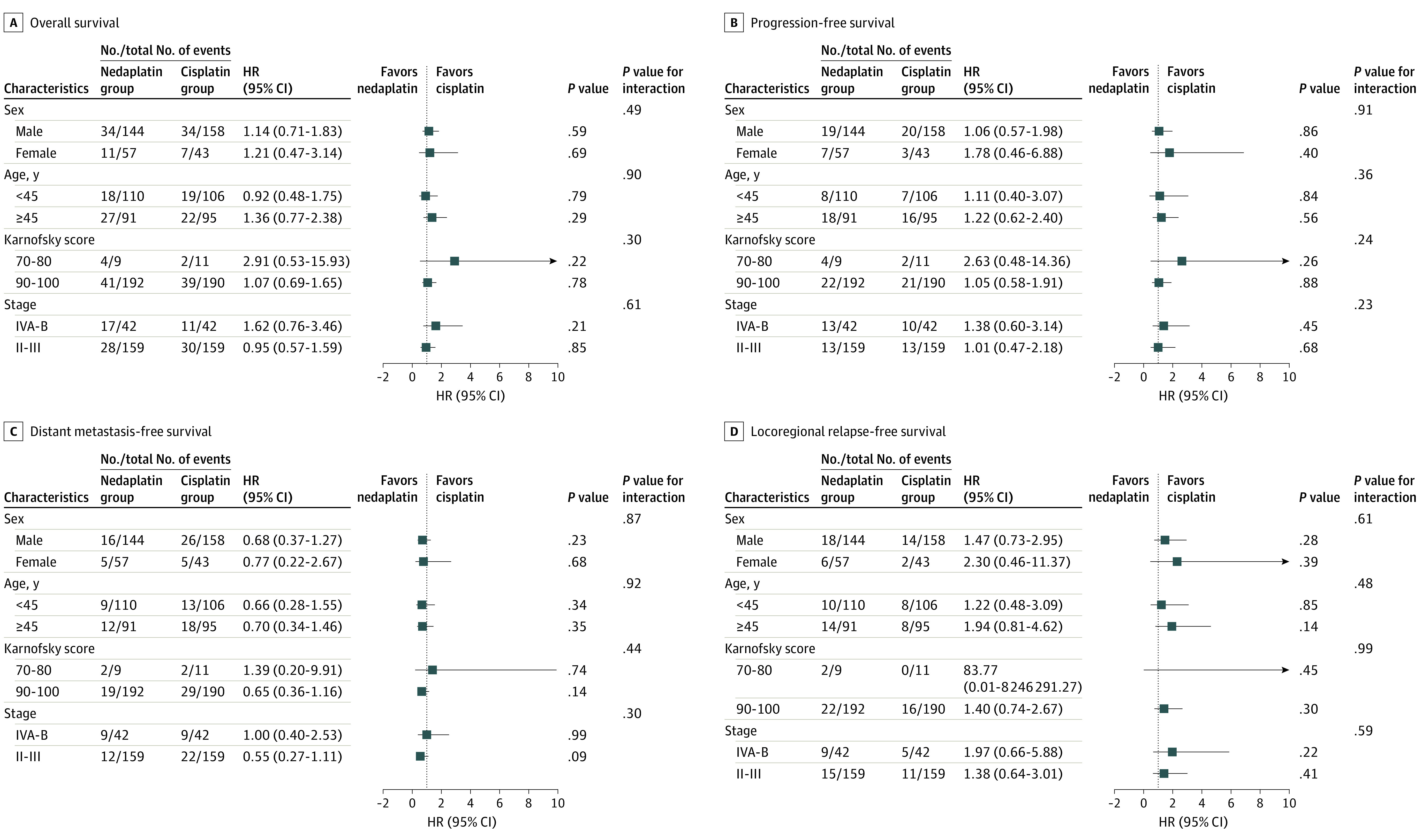
Treatment Effects on Survival Within Subgroups The number of events and the number of patients are shown by study arm. Hazard ratios (HRs) and 95% CIs were calculated in a univariate Cox proportional hazards regression model; interaction and stratified analyses were conducted according to sex, age, Karnofsky score, and stage.

We performed subgroup analyses for OS, PFS, DMFS, and LRFS in patients stratified by the following covariates: sex (male or female), age (45 or ≥45 years), Karnofsky Performance Status score (70-80 or 90-100), and disease stage (II-III or IVA-B). No interactions between these covariates and treatment were observed (male sex: HR, 1.14; 95% CI, 0.71-1.83; female sex: HR, 1.21; 95% CI, 0.47-3.14; *P* = .49 for interaction; age <45 years: HR, 0.92; 95% CI, 0.48-1.75; age ≥45 years: HR, 1.36; 95% CI, 0.77-2.38; *P* = .90 for interaction; Karnofsky Performance Status score of 70-80: HR, 2.91; 95% CI, 0.53-15.93; Karnofsky Performance Status score of 90-100: HR, 1.07; 95% CI, 0.69-1.65; *P* = .30 for interaction; stage IVA-B disease: HR, 1.62; 95% CI, 0.76-3.46; stage II-III disease: HR, 0.95; 95% CI, 0.57-1.59; *P* = .61 for interaction) (Figure 3), suggesting that the noninferiority of nedaplatin-based CCRT did not differ among specific populations.

### Toxic Effects

Toxic effects were assessed in the safety population, which included 398 patients who received CCRT. Publication of the early results of this trial included details of adverse events during treatment and the quality of life of patients.^9^ In this long-term analysis, we evaluated the late adverse events that occurred after the concurrent chemotherapy with nedaplatin or cisplatin (Table 2). Patients in the cisplatin group experienced a significantly higher incidence of any grade or grade 3 to 4 auditory toxic effects vs patients in the nedaplatin group (grade ≥1: 82/200 [41.0%] vs 112/198 [56.6%], *P* = .002; grade 3-4: 21 [10.5%] vs 35 [17.7%], *P* = .04). We further analyzed the cumulative incidence for auditory toxic effects, and the results indicate patients in the cisplatin group had a higher cumulative incidence of grade 3 to 4 auditory toxic effects vs patients in the nedaplatin group (19.9% vs 12.0%, *P* = .42) (eFigure 2 in Supplement 2). Moreover, the post hoc regression analysis performed to examine the association between patient factors also demonstrated patients in the nedaplatin group have a lower risk of auditory toxic effects (odds ratio, 0.51; 95% CI, 0.28-0.93; *P* = .03) (eTable 3 in Supplement 2).

**Table 2.** Late Adverse Events in the Safety Population^a^

Toxic effect	No. (%) of adverse events						*P* value^b^	
	Nedaplatin group (n = 200)			Cisplatin group (n = 198)			For events grade ≥1	For events grade ≥3
	Grade 1-2	Grade 3	Grade 4	Grade 1-2	Grade 3	Grade 4		
Auditory	61 (30.5)	13 (6.5)	8 (4.0)	77 (38.9)	24 (12.1)	11 (5.6)	.002	.04
Trismus	24 (12.0)	1 (0.5)	0	36 (18.2)	0	0	.12	>.99
Dysphagia	31 (15.5)	0	0	33 (16.7)	0	0	.75	NA
Skin	74 (37.0)	0	1 (0.5)	71 (35.9)	0	0	.73	>.99
Subcutaneous soft tissue	67 (33.5)	5 (2.5)	0	71 (35.9)	5 (2.5)	0	.62	>.99
Dry mouth	107 (53.5)	19 (9.5)	1 (0.5)	112 (56.6)	19 (9.6)	0	.58	.89
Cranial neuropathy	40 (20.0)	3 (1.5)	1 (0.5)	47 (23.7)	2 (1.0)	0	.52	.69
Peripheral neuropathy	27 (13.5)	0	0	35 (17.8)	1 (0.5)	0	.20	.497
Endocrine dysfunction	7 (3.5)	1 (0.5)	0	6 (3.0)	0	0	.80	>.99
Temporal lobe necrosis	28 (14.0)	2 (1.0)	0	33 (16.7)	2 (1.0)	0	.47	>.99

Abbreviation: NA not applicable (no grade 3 and grade 4 adverse events in both groups).

^a^ Late adverse events defined as toxic effects that occurred 6 months after completion of radiotherapy.

^b^ *P* values were calculated using the χ^2^ test. No grade 5 late adverse events were found during follow-up.

No significant intergroup differences were found in late toxic effects of trismus, dysphagia, skin, subcutaneous soft tissue, dry mouth, peripheral neuropathy, cranial neuropathy, endocrine dysfunction, and temporal lobe necrosis, regardless of grade (1-2 or 3-4).

## Discussion

In this secondary analysis of a randomized clinical trial, the 5-year survival results were consistent with those at 2 years.^17^ Patients from the nedaplatin group achieved comparable 5-year PFS, OS, DMFS, and LRFS rates as those in the cisplatin group. The comparison groups in this study were well balanced in terms of patient characteristics, tumor factors, and treatment parameters. Nedaplatin-based CCRT had noninferior therapeutic efficacy to cisplatin-based CCRT in patients with stage II to IVB NPC. Of foremost importance, patients in the nedaplatin group had fewer late toxic effects.

Chemotherapy-induced toxic effects have always been a major concern in the treatment of NPC. Thus, screening effective and low-toxicity antitumor drugs is the common goal of oncologists. Nedaplatin is a second-generation cisplatin derivative that was developed to enhance the antitumor effects of cisplatin and alleviate renal dysfunction, hearing deficit, and gastrointestinal toxic effects. Thus, nedaplatin was a promising substitute drug for cisplatin.^18,19,20^

A phase 2 study conducted by Zheng et al^15^ found that nedaplatin (100 mg/m^2^) and fluorouracil induction chemotherapy, followed by IMRT concomitant with nedaplatin, is an effective and safe treatment for NPC. A subsequent prospective study^14,15^ of nedaplatin-based concurrent chemoradiotherapy found that nedaplatin CCRT administered every 3 weeks for 3 cycles resulted in mild adverse effects and good patient adherence.

In this study, the survival rates in the 2 treatment arms for the intention-to-treat or per-protocol populations were almost identical during the first 2 years and then steadily extended with time; survival rates in the nedaplatin group were noninferior to those of the cisplatin group at 5 years (PFS: 79.8% vs 81.4%; *P* = .002 for noninferiority; OS: 88.8% vs 89.4%; log-rank *P* = .63). Patients who received cisplatin-based CCRT had relatively higher distant metastasis relapse rates compared with patients who received nedaplatin-based CCRT. This finding might be partially explained by the fact that nedaplatin had a radiosensitizing effect and was effective in patients who were refractory to cisplatin-based chemotherapy.^12,21^

The acceptable width of the margin of noninferiority is a controversial aspect in the design of noninferiority studies. Wide margins allow smaller sample sizes to conclude noninferiority, but if a margin is too wide, a conclusion of noninferiority could be clinically irrelevant or ethically inappropriate. We set an approximate value of 10% as a noninferiority margin in this trial. In the IMRT era, CCRT has been established as the backbone treatment for locoregionally advanced NPC, and only a few patients with locoregionally advanced NPC underwent radiotherapy alone. Prospective randomized clinical trials on CCRT vs radiotherapy in the IMRT era are limited. In a previously published individual patient data network meta-analysis,^22^ the absolute benefit at 5 years of PFS was 10% for CCRT vs radiotherapy alone. However, this meta-analysis,^22^ which contains trials in both the 2-dimensional chemoradiotherapy and IMRT eras, found that the CCRT regimen was different, including cisplatin, carboplatin, oxaliplatin, uracil tegafur, and fluorouracil. The lack of survival probabilities for CCRT vs radiotherapy from randomized clinical trials in the IMRT era made us refer to previously published clinical trials of CCRT vs radiotherapy alone, which reported a PFS benefit of approximately 18% to 40% at 2 years from the addition of concurrent cisplatin chemotherapy.^8,23^ Among the cancer trials that reported a prespecified noninferiority margin, the median value was 12.5% (range, 4%-25%).^24^ In addition, Pong et al^25^ found that the overall median noninferiority margin was an absolute risk difference of 10% (IQR, 7.5%-13.8%) in oncologic trials. Two recently published studies^26,27^ on NPC also adopted 10% as the noninferiority margin. This margin of reduced efficacy of cisplatin was regarded as clinically acceptable in view of the expected reduced toxic effects, increased quality of life, and more convenient schedules of administration of nedaplatin.

With regard to acute toxic effects, this study’s initial analysis^17^ found that patients treated with cisplatin-based CCRT had a higher incidence of grade 3 or 4 vomiting (18% vs 6%, *P* < .001), nausea (9% vs 2%, *P* = .002), and anorexia (27% vs 13%, *P* < .001) than those treated with nedaplatin-based CCRT. However, patients in the nedaplatin group had a relatively higher frequency of grade 3 or 4 thrombocytopenia compared with the cisplatin group (6% vs 2%, *P* = .06). For late toxic effects, the 2-year results showed a higher incidence of grade 3 or 4 late auditory toxic effects in the cisplatin group compared with the nedaplatin group (6% vs 2%, *P* = .03).^17^ In this long-term analysis, a significantly higher incidence of any grade or grade 3 and 4 auditory toxic effects was also observed in the cisplatin group compared with that in the nedaplatin group (35 [17.7%] vs 21 [10.5%], *P* = .04). The incidences of trismus, dysphagia, dry mouth, peripheral neuropathy, cranial neuropathy, endocrine dysfunction, temporal lobe necrosis, and toxic effects of skin and subcutaneous soft tissue were all comparable between the 2 groups. Compared with the cisplatin-based regimen, the nedaplatin-based regimen was associated with minimal nephrotoxic effects and gastrointestinal and hearing toxic effects.

The cost-effectiveness analysis by Liao et al^28^ indicated that nedaplatin is more cost-ineffective compared with cisplatin for CCRT in locoregionally advanced NPC in China. An ongoing phase 3 clinical trial, which replaced cisplatin with nedaplatin or fluorouracil with capecitabine during the induction chemotherapy and CCRT phases may help confirm the efficacy and toxic effects of nedaplatin.^2^

### Strengths and Limitations

This study has several strengths, including the large sample size, long-term follow-up, and consistency of the results at different end points. Nevertheless, this study also has several limitations. First, pretreatment measurements of prognostic biomarkers, such as plasma EBV-DNA, are not mandatory and thus were not included as a patient selection criterion. Second, this trial was initially designed to enroll patients from 4 centers; however, 2 centers withdrew from this trial after study initiation. Third, the patient randomization allocation procedure was performed manually, which is more prone to allocation error than a central random system (telephone or internet based). Fourth, as mentioned in the early report^17^ of this trial, participants in the nedaplatin group had lower adherence, with 3 complete treatment cycles compared with participants in the cisplatin group, which could be a confounder when analyzing intergroup differences in toxic effects. Fifth, the primary study end point was not centrally reviewed. Sixth, the tumor volumes were not calculated before randomization. Seventh, the high fractionation of 2.33 Gy might be a confounder and lead to more severe late toxic effects. Eighth, whether these results could apply to patients from non-endemic areas remains to be confirmed in future studies.

## Conclusions

This long-term secondary analysis confirms that nedaplatin-based CCRT could be an alternative to cisplatin-based CCRT as doublet therapy for stage II to IVB NPC. Nedaplatin-based CCRT incurred a significant decrease in the overall late toxic effect rate. Further investigations are needed to explore the potential of nedaplatin in combination drug chemotherapy in the induction or adjuvant phase. Our findings could potentially help widen the choice of concurrent chemotherapeutic regimens for patients with NPC.

## Notes

Supplement 1.
Trial Protocol

Supplement 2. **eTable 1.** Comparison of Survival Outcomes Between the Nedaplatin and Cisplatin Groups **eTable 2.** The 5-Year Survival Rate for the Intention-to-Treat and Per-Protocol Analysis **eTable 3.** Factors Associated With Grade 3–4 Auditory/Hearing Toxicities in the Safety Populations **eFigure 1.** Progression-Free Survival (A), Overall Survival (B), Cumulative Incidence of Distant Metastasis (C), and Cumulative Incidence of Locoregional Relapse (D) in the Per-Protocol Populations **eFigure 2.** Cumulative Incidence of Grade 3–4 Auditory/Hearing Toxicities in the Safety Populations With Regards to Different Treatment Group

Supplement 3.
Data Sharing Statement
