# Supplementary material for: Effect of Concurrent Chemoradiotherapy With Nedaplatin vs Cisplatin on the Long-term Outcomes of Survival and Toxic Effects Among Patients With Stage II to IVB Nasopharyngeal Carcinoma: A 5-Year Follow-up Secondary Analysis of a Randomized Clinical Trial
Source: JAMA Netw Open. 2021 Dec 20;4(12):e2138470. doi: 10.1001/jamanetworkopen.2021.38470 (PMC8689390; doi:10.1001/jamanetworkopen.2021.38470)
Supplement: Supplement 1. — Trial Protocol [file jamanetwopen-e2138470-s001.pdf]

# A Randomized Phase III Non-inferiority Study of Concurrent Chemoradiotherapy with Nedaplatin versus Cisplatin in Staged II-IVb Nasopharyngeal Carcinoma

## FINAL PROTOCOL
**Version: December 20, 2011**


**Principle Investigator: Dr. Hai-Qiang Mai**

**Sponsor: Sun Yat-sen University Cancer Center**

**Collaborators:**

Guangzhou Medical University

Study Coordinator: Dr. Dongping Chen

Meizhou City Hospital of Guangdong Province

Study Coordinator: Dr. Jun Xiong

The First Affiliated Hospital of Guangdong Pharmaceutical University

Study Coordinator: Dr. Xicheng Wang

# CONTENTS

**SCHEMA**

**1.0 Background**

**2.0 Objectives**

**3.0 Subject Enrollment**

3.1 Eligibility Criteria
3.2 Exclusion Criteria
3.3 Criteria for Removal from Protocol Treatment

**4.0 Treatment Plan**

4.1 Chemotherapy
4.2 Radiotherapy
4.3 Salvage Therapy

**5.0 Drug Information**

5.1 Cisplatin
5.2 Nedaplatin

**6.0 Observation and assessment**

6.1 Before Treatment
6.2 During Treatment

**7.0 Follow up and record of events**

**8.0 Security Measures and Quality Control**

**9.0 Statistical Analysis**

**10.0 Ethical Considerations**

**11.0 Schedule**

**12.0 BIBLIOGRAPHY**

**Appendix I**

**Appendix II**

**Appendix III**

**SCHEMA**

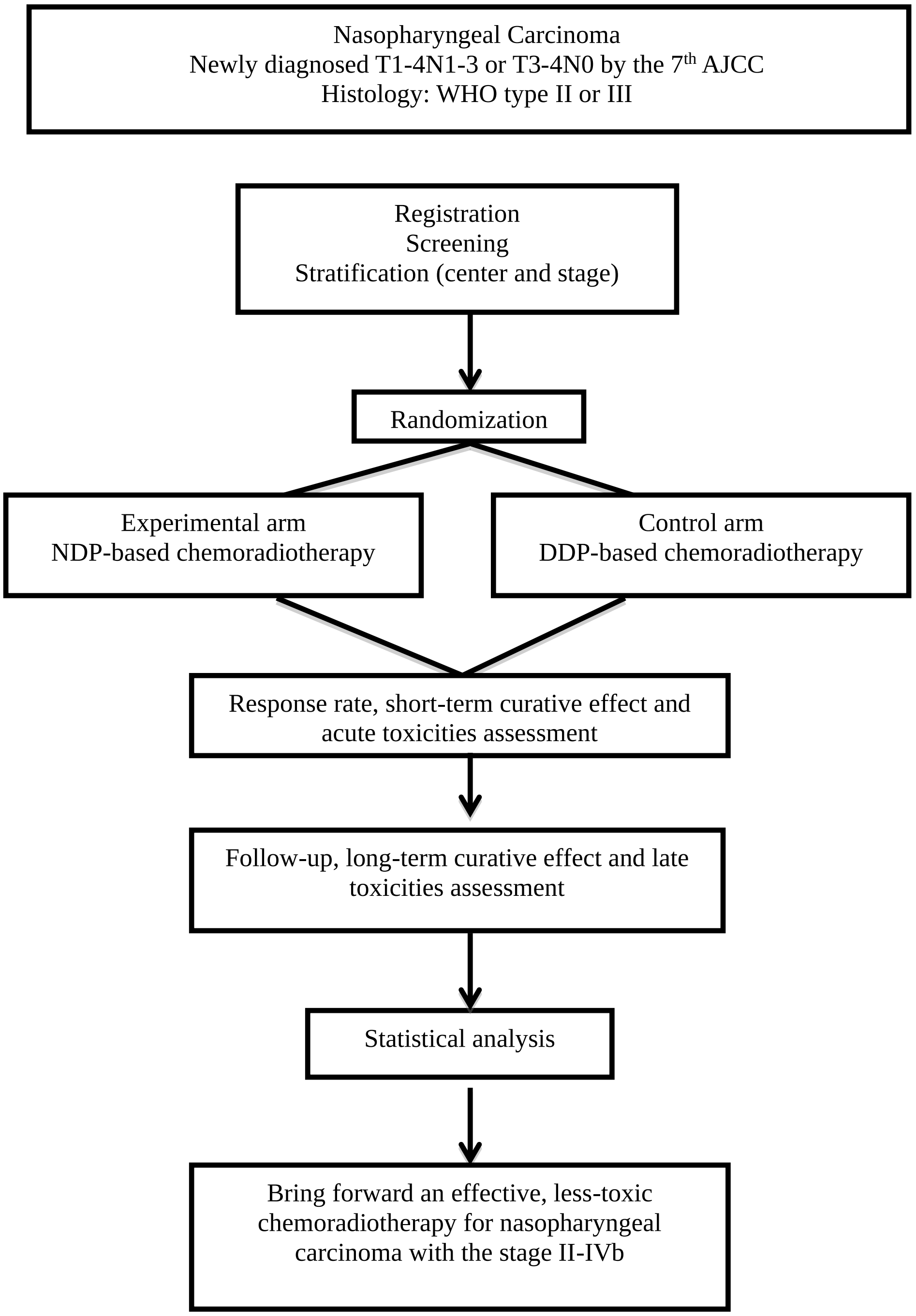


*NDP=Nedaplatin; DDP=Cisplatin

## 1.0Background

Nasopharyngeal carcinoma (NPC) is one of the most common malignant tumors in China, which is especially endemic in Southern China and Hong Kong. Radiation is the major therapy for NPC. The 5-year survival rate in stage I NPC patients goes up to 90% while treated with radiotherapy alone. However, due to the covert location of nasopharynx, many patients are asymptomatic and 70% of them have progressed to locoregionally advanced staged NPC at the time of confirmed diagnosis, and the 5-year survival rate only ranges from 30%-50% under radiotherapy[1].Therefore, it has become a research focus that how to reduce locoregional recurrence and distant metastasis in locoregionally advanced staged NPC and further establish a multi-modality treatment mode to improve overall survival.

Concurrent chemoradiotherapy (CCRT) is the standard treatment for locoregionally advanced staged NPC, it has showed its superiority to improve OS, as well as promote locoregional control and suppress metastasis[2-7]. Our previous study proved for the first time that CCRT was beneficial to significantly improve OS rate, progression-free (PFS) rate and distant disease-free survival (DDFS) rate in stage II NPC patients[8]. Similar results were also showed in 3 published meta-analysis that the survival benefits of locoregionally advanced NPC patients mainly resulted from CCRT rather than other combinations of radiotherapy and chemotherapy[9-11].

Cisplatin is a major chemotherapy medication for locoregionally advanced NPC. However, patients' tolerance is greatly impaired and quality of life threatened due to the severe side effects such as gastrointestinal reactions, nephrotoxicity, ototoxicity, neurotoxicity. Because of the side effects and the need of hydrotherapy while given high dose, it is demanding for patients to complete the full-cycle chemotherapy. According to the phase III clinical trials conducted in America, Singapore and Hong Kong, only 50%-70% of patients were able to complete the concurrent cisplatin-based chemoradiotherapy for 3 cycles, with dose intensity of cisplatin only 24-30mg/m$^2$ per week[2,6,7]. The compliance of patients was rather unsatisfactory under the treatment of weekly regimen. A Hong Kong randomized trial demonstrated that only 44% of patients completed the 6-cycle chemotherapy, and the dose intensity of cisplatin was far from the expected 40mg/m$^2$ weekly[3]. In spite of the improvement of OS rate with the application of cisplatin, it significantly increases the acute and late toxicities of radiation. It was observed in NPC-9901 trial[7] from Hong Kong that the CRT arm had significantly more acute toxicities (84% vs. 53%, $P<0.01$) and late toxicities compared with the RT arm. The majority of toxicities were grade 3 in severity with increased incidence of otologic toxicities (14% vs. 8%), peripheral neuropathy (2% vs. 0) and endocrine dysfunction (4% vs. 1%). Consequently, it is of great necessity to find an effective, less toxic and well-tolerated platinum-based medication, in which way patients can complete the 3-cycle chemotherapy as scheduled and attain better therapeutic effect.

The substitute for cisplatin for NPC is in urgent need, while the application of carboplatin or oxaliplatin is not optimal for NPC. Carboplatin is the 2$^{nd}$ generation platinum-based medication that possesses the characteristic of less nephrotoxicity and milder gastrointestinal reactions. However, carboplatin was found inferior to cisplatin in terms of therapeutic effect of CCRT for NPC patients[12-14]. Moreover, the sensitivity of carboplatin was reduced by radiation in vitro[15], which also showed the inadequacy of carboplatin in chemoradiation. Oxaliplatin is the 3$^{rd}$ generation platinum compounds. Zhang et al.[5]compared standard radiotherapy with or without weekly oxaliplatin in treatment of locally advanced NPC, and the 2-year OS rate in the experimental arm reached 100% (v.s.77% in the control arm) and no severe oxaliplatin-related side effects were observed. Nevertheless, the application of oxaliplatin in NPC is still limited because of its high expense. Besides, the main indication of oxaliplatin is colorectal carcinoma rather than NPC.

Nedaplatin is a new 2$^{nd}$generation platinum-based drug that functions by interfering with DNA replication. It has been confirmed that the types of combined bases in are identical to those observed in cisplatin while the water solubility of nedaplatin increases by 10 times compared with cisplatin[16].The major adverse effect is myelosuppression, such as thrombocytopenia, which often appears to be grade 1-3 with quick recovery[17].Served as a new platinum derivative, nedaplatin not only has curative effect but also reduces the incidence of severe gastroenteritis and nephropathy, thus bringing more survival benefits and better quality of life to patients. Studies indicated that the efficacy of nedaplatin equaled or even exceeded that of cisplatin in treatment with head-neck tumors and esophageal squamous cell carcinoma. The application of nedaplatin has already been mentioned by NCCN guidelines for esophagus cancer in 2007. As for treating head and neck tumors, it was found to have a response rate of over 40% (vs. 30% response rate of cisplatin) and much slighter nephrotoxicity and gastrointestinal reactions[18-19].Moreover, its sensitivity was enhanced by radiation in vitro[15] while it also showed its potential as a radiosensitizing agent.

Nedaplatin showed therapeutic effect in treating NPC. A phase II clinical trial enrolled 60 patients with locoregionally advanced NPC, and they were treated with induction chemotherapy of Nedaplatin+5-FU followed by concomitant nedaplatin -based chemoradiation (100 mg/m$^2$, every 3 weeks

for 3 cycles). The treatment was proved to be effective and safe, with the 3-year PFS 75.0% and OS 85.5%[20]. Nedaplatin has good prospects for the treatment of NPC, but high-grade evidence-based researches are still absent. We consider nedaplatin a potential first-line drug for concomitant chemoradiation of NPC, which need to be proved by large-sample randomized clinical trials.

Therefore, we are planning a non-inferiority multi-center randomized clinical trial, and the primary outcome measures progression-free survival. The expected result is that nedaplatin is noninferior than cisplatin in concurrent chemoradiation, and its side effects are fewer and milder, thus an improvement in patients' compliance and quality of life will be obtained. If proved, high-grade evidence will be provided for the application of nedaplatin to concurrent chemoradiatherapy of NPC, which is of great value to optimize therapeutic options for concomitant chemotherapy of NPC.

## 2.0 Objectives

### 2.1 The primary objectives

To determine whether CCRT with nedaplatin will result in noninferior progression-free survival as compared with those patients receiving cisplatin in staged II-IVb nasopharyngeal carcinoma.

### 2.2 Secondary objectives

To compare overall survival, Locoregional relapse-free survival, distant metastasis-free survival, response rate, toxicity profile, compliance of the treatment, quality of life and cost effectiveness between nedaplatin and cisplatin arms.

## 3.0 Subject Enrollment

### 3.1Eligibility Criteria

a. Patients with histologically confirmed non-keratinizing nasopharyngeal carcinoma (including differentiated type and undifferentiated type, WHO II or III)
b. Original clinical staged as T1-4N1-3 or T3-4N0 (according to the 7th AJCC edition)
c. No evidence of distant metastasis (M0).
d. Age between 18-65 years
e. WBC $\geq 4 \times 10^9$/L , PLT $\geq 100 \times 10^9$/L, HGB $\geq$90g/L
f. With normal liver function test (TBil, ALT and AST≤2.0*ULN)
g. With normal renal function test (Ccr≥60 ml/min or Creatinine≤1.5*ULN)
h. Satisfactory performance status: Karnofsky scale (KPS) ≥70
i. Patients must be informed of the investigational nature of this study and give written informed consent.

### 3.2 Exclusion Criteria

a. Patients with histologically confirmed ketatinizing squamous cell carcinoma (WHO I)
b. Age >65 or < 18 years
c. Treatment with palliative intent.
d. Prior malignancy except adequately treated basal cell or squamous cell skin cancer, in situ cervical cancer.
e. Prior chemotherapy or surgery (except diagnostic) to primary tumor or nodes.
f. History of previous RT (except for non-melanomatous skin cancers outside intended RT treatment volume).
g. Pregnancy or lactation (consider pregnancy test in women of child-bearing age and emphasize effective contraception during the treatment period).
h. Any severe intercurrent disease, which may bring unacceptable risk or affect the compliance of the trial, for example, unstable cardiac disease requiring treatment, acute exacerbation of chronic obstructive pulmonary disease or other respiratory illness requiring admission to hospital, renal disease, chronic hepatitis, diabetes with poor control (fasting plasma glucose >1.5×ULN), and mental disturbance.

### 3.3 Criteria for Removal from Protocol Treatment

a. Disease progression
b. Unacceptable toxicity. The reason(s) must be recorded.

c. Intercurrent diseases which may affect assessments of clinical status to a significant degree and require discontinuation of drug, or both.
d. The patient may withdraw from the study at any time for any reason. The reason should be recorded.

## 4.0 Treatment Plan

### 4.1 Chemotherapy

**4.1.1 Nedaplatin-based chemotherapy regimen (experimental arm)**:

Nedaplatin is given 100mg/$m^2$ IV every 3 weeks (on day 1, 22, 43) for 3 cycles during the RT phase.

**4.1.2 Cisplatin-based chemotherapy regimen (control arm)**:

Cisplatin is given 100mg/$m^2$ IV every 3 weeks (on day 1, 22, 43) for 3 cycles during the RT phase.

***NOTE:***

*Given the consideration that chemotherapy reduces the incidence of distant metastasis, if only 2 cycles of concurrent chemotherapy are completed during the RT phase, then the third cycle of chemotherapy will be given within a week after completion of RT as planned.

*If unpredicted situations such as allergy arise in the arm of nedaplatin, cisplatin will be given as salvage treatment for concurrent chemotherapy.

**4.1.3 Administration:**

**4.1.3.1Cisplatin Administration Guidelines:**

**4.1.3.1.1** Follow local hydration protocols to prevent/minimize nephrotoxicity. Pre-hydration (at least 12 hours prior to cisplatin administration, D0) and post-hydration (D1-3 of cisplatin injection) with over 2,000ml of fluid infused each time are both needed. The use of mannitol (D1) and furosemide (D1) is essential for it helps to reduce cisplatin-related nephrotoxicity by diuresis. 24h urinary output should be recorded (D0-D3) and urinalysis performed (D2-D3). Electrolytes and serum creatinine should be monitored during treatment, and a follow-up creatinine within 24-48 hours of the cisplatin dose is recommended.

**4.1.3.1.2**Patients will be treated with antiemetic agents such as the 5-HT3-receptor antagonist, dexamethasone and metoclopramide before cisplatin chemotherapy on day 1, thereafter on days 2-3.

**4.1.3.2 Nedaplatin Administration Guidelines:**

Nedaplatin will be dissolved in 0.9% sodium chloride injection first, and added to 500ml NS before intravenous infusion. The injection should be lasted for more than 1h, and moderate post hydration therapy is also needed through an intravenous drip of 1,000ml NS (D1).

Patients will be treated with antiemetic agents such as the 5-HT3-receptor antagonist, dexamethasone and metoclopramide before nedaplatin chemotherapy on day 1.

**4.1.4Dosage Adjustments**

**4.1.4.1** Patients will be examined and graded for subjective/objective evidence of developing toxicity according to the CTCAE, v. 3.0 each day that chemotherapy is administered and weekly while receiving radiotherapy.

**4.1.4.2** There will be no dose escalation for concurrent nedplatin or cisplatin.

**4.1.4.3** Nedaplatin or cisplatin dose modifications for chemotherapy were intended to be permanent (ie, if a patient's dose was reduced to 80 mg/m², it remained at the reduced dose for the duration of their treatment).

**4.1.4.4** Hematologic growth factors for neutropenia or anemia are not allowed during concurrent chemoradiotherapy.

**4.1.4.5** Chemotherapy must not be administered until the absolute neutrophil count ≥ 1,500 and platelets count are ≥ 100,000.

| Cisplatin Dose Levels | | |
|---|---|---|
| **-2** | **-1** | Starting Dose |
| 60 mg/$m^2$ | 80 mg/$m^2$ | 100 mg/$m^2$ |

| Nedapaltin Dose Levels | | |
|---|---|---|
| **-2** | **-1** | Starting Dose |
| 60 mg/$m^2$ | 80 mg/$m^2$ | 100 mg/m2 |

**4.1.4.1 Dose Adjustment for Hematological Toxicity**

| Absolute Neutrophil Count | | Platelet Count | Cisplatin or Nedaplatin Dose Adjustment |
|---|---|---|---|
| ≥1,500 | and | ≥75,000 | full dose |
| 1,000-1,499 | and/or | 50,000-74,999 | Decrease 1 level |
| <1,000 | and/or | <50,000 | Decrease 2 level* |

*If the patient is already at dose level 1, then decrease to dose level 2. If the patient is already at level 2,

then discontinue concurrent cisplatin or nedaplatin.

### 4.1.4.2 Dose Adjustment for Non-hematologic Adverse Events

**4.1.4.2.1**Neutropenic fever:

Temperature of 38.5° C with ANC < 1000 is an expected potential complication of concurrent chemotherapy and radiotherapy. If neutropenic fever is noted, the chemotherapy dose reduction will be determined by the weekly blood counts.

**4.1.4.2.2**Allergic reaction/hypersensitivity

Severe hypersensitivity reactions (grade 3 or more) related to nedaplatin required immediate discontinuation of chemotherapy. Patients with a history of severe hypersensitivity reactions should not be rechallenged with nedaplatin. Cisplatin will be given as salvage treatment for concurrent chemotherapy.

**4.1.4.2.3**Renal adverse events:

Dose will be modified based on the creatinine clearance immediately prior to each cisplatin or nedapaltin dose. The creatinine clearance was calculated with Cockcroft formulation. The dose modified as indicated below:

| Creatinine Clearance | Cisplatin or Nedaplatin Dose Adjustment |
|---|---|
| ≥60 ml/min | Full dose |
| 40-60 ml/min | Decrease 1 level |
| <40 ml/min | Hold drug* |

*Cisplatin or nedaplatin must not be administered until creatinine clearance ≥60 ml/min. If creatinine clearance remains < 40 ml/min, the patient should not receive additional cisplatin or nedaplatin.

**4.1.4.2.4**Neurologic Adverse Events

The dosage of cisplatin or nedaplatin decreases 1 level when patients suffer from neurotoxicity of grade 2. Patients with neurotoxicity of grade 3 or more are withdrawn from the study.

**4.1.4.2.5**Ototoxicity

Should patients develop clinical evidence of ototoxicity, further audiometric evaluation is required. Patients with clinically significant hearing loss must not receive additional cisplatin or nedaplatin. Cisplatin or nedapaltin should be held for grade 3 hearing loss that has primarily a neurological basis; for grade 2 hearing loss with primarily a neurological basis, decrease 1 level.

**4.1.4.2.56**Patients should be cautioned on the need for contraception during the treatment period.

**4.1.4.2.67**Any death possibly attributed to drug therapy must be reported to the study coordinator and central office

## 4.2 Radiotherapy

Patients will be examined and graded for subjective/objective evidence of acute toxicities according to the CTCAE toxicity criteria.

**4.2.1RT adjustments for non-hematological toxicity:**

Side effects of RT may include mucositis and skin reaction. The investigator will manage these conditions according to clinical practice at the institution. We allowed no radiotherapy dose modifications. Treatment interruptions were allowed if symptomatic mucositis or skin reactions occurred that, in the judgment of the attending clinician, warranted a break. The treatment was completed according to protocol for treatment breaks up to and including 14 days. If the break exceeded 14 days, the patient was removed from protocol treatment, completing treatment at the discretion of his or her physician but followed up and included in the analysis.

**4.2.2 RT adjustments for hematological toxicity:**

RT will be withheld until ANC >500 and platelet >25,000.

**4.2.3 Target Volume Determination for IMRT**

Radiotherapy must be given with Intensity Modulated RT (IMRT) techniques. Target volumes were defined in accordance with the International Commission on Radiation Units and Measurements (ICRU) reports 50 and 62. The principle of target volume determination for IMRT and prescribed dose and fractionation are as follows:

**Principle of Target Volume Determination for IMRT**

| Term | Definition | Note |
|---|---|---|
| Gross tumor volume (GTV) | The gross tumor determined by physical examination, imaging (including MRI and PET/CT, if available) and endoscopic findings, including GTVnx and GTVnd. | |
| GTVnx | Included the sum of the primary tumor volume and the enlarged retropharyngeal nodes | |
| GTVnd | The volume of clinically involved gross lymph nodes | |
| CTV1 | GTVnx plus an additional anterior, superior, inferior and lateral margin of 5mm to 1cm, and an additional posterior margin of 2mm to 3mm (the range of extension is determined by adjacent | the volume should also include the entire mucosal stratum and 5mm of submucosal stratum of nasopharynx |

| | | |
|---|---|---|
| | structural characteristics) | |
| CTV2* | CTV1 plus an additional anterior, superior, inferior and lateral margin of 5mm to 10mm, and an additional posterior margin of 2mm to 3mm (the range of extension is determined by adjacent structural characteristics), and GTVnd plus possible tumor-draining lymph node groups that are at risk of potential microscopic spread of disease | The range of prophylactic neck radiation should extend from involved lymph node groups to 1 or 2 adjacent groups |
| PTV | PTVnx, PTVnd, PTV1, PTV2 respectively refers to GTVnx, GTVnd, CTV1, CTV2 plus an additional margin, with an anterior, superior, inferior, lateral extension of 5mm, and a posterior extension of 3mm in general | |
| Organs at risk | brainstem, temporal lobe, lens, eyeballs, optic nerves, optic chiasm, pituitarium, parotid gland, temporomandibular joint, mandible, larynx, oral cavity, salivary gland, inner and middle ear | Organs can be added or removed according to actual situations |

*Level Ib was electively irradiated if: (1) level Ib lymph nodes (LNs) were involved, (2) level IIa LNs had extracapsular extension or diameter ⩾ 3 cm, (3) there was extensive nodal disease on the ipsilateral neck, (4) the soft or hard palate, oral cavity, or ipsilateral nasal cavity was grossly involved.

### 4.2.4 Prescribed dose and fractionation:

All patients will be treated with IMRT using simultaneously integrated boost, 5 fractions per week. The prescribed dose was 66–70 Gy, 64–70 Gy, 60–62 Gy, and 54–56 Gy, in 30–33 fractions, for the PTVs derived from GTVnx, GTVnd, CTV1, and CTV2, respectively.

### 4.2.5 Normal tissue dose constraints

**Normal tissue dose constraints Structure**

| Structure | Dose constraints |
|---|---|
| Spinal cord | Dmax* ⩽ 45 Gy |
| Spinal cord_PRV | D1†≤ 54 Gy |
| Brain stem | Dmax ≤ 54 Gy |
| Brain stem_PRV | D1 ≤ 60 Gy |
| Optic nerves | Dmax ≤ 54 Gy |
| Optic nerves_PRV | D1 ≤ 60 Gy |
| Optic chiasm | Dmax ≤ 54 Gy |
| Optic chiasm_PRV | D1 ≤ 60 Gy |
| Temporal lobe | Dmax ≤ 60 Gy |
| Temporal lobe_PRV | D1 ≤ 65 Gy |
| Lens | Dmean‡ < 8 Gy |
| Pituitary | Dmax < 60 Gy |
| Eyes | Dmean < 35 Gy |
| Mandible | Dmax < 70 Gy |
| Temporomandibular Joint | Dmax < 70 Gy |
| Parotid | Dmean < 26 Gy |
| Parotid | V30§ < 50% |
| Cochlea | Dmean < 50 Gy |
| Larynx | Dmean < 45 Gy |

PRV = planning organ at risk volume.
* Maximum point dose to the target volume.
† Dose received by 1% of the target volume.
‡ Mean dose to the target volume.
§ At least 50% of the gland will receive <30 Gy (should be achieved in at least one gland)

### 4.3 Salvage Therapy

Nasopharyngeal surgery, neck dissections, secondary radiotherapy or chemotherapy may be given to patients with relapse or metastasis after treatment.

## 5.0 Drug Information

### 5.1Cisplatin

**5.1.1**Producted by Hospira Australia Pty Ltd.

**5.1.2Pharmacology and Pharmacokinetics:** The dominant mode of action of cisplatin appears to be inhibition of the incorporation of DNA precursors, although protein and RNA synthesis are also inhibited. Plasma levels of cisplatin decay in a biphasic mode with an initial half-life of 25 to 49 minutes, and a secondary phase ranging from 58 to 73 hours. This prolonged phase is due to protein binding which exceeds 90% of the radioactivity in the second phase. Urinary excretion is incomplete with only 27 to 45% of the radioactivity excreted in the first 5 days. The initial fractions of radioactivity are largely unchanged drugs. Although this drug seems to act as an alkylating agent, there are data to indicate that its mode and sites of action are different from those of nitrogen mustard and the standard alkylating agents.

**5.1.3Toxicity:** Human toxicity includes nausea, vomiting, anorexia, loss of taste, renal toxicity (with an elevation of BUN, creatinine, and impairment of endogenous creatinine clearance, as well as renal tubular damage which appears to be transient), ototoxicity (with hearing loss which initially is in the high-frequency range, as well as tinnitus), peripheral neuropathy, allergic reactions, and hyperuricemia. Much more severe and prolonged toxicity has been observed in patients with abnormal or obstructed urinary excretory tracts. Myelosuppression, often with delayed erythrosuppression, is expected. In the high-dose treatment regimen with osmotic diuresis, the nadir of white cells and platelets occurred regularly at about 2 weeks with recovery generally at about 3 weeks after the initiation of therapy. The occurrence of acute leukemia has been reported rarely in patients treated with anthracycline/alkylate or combination chemotherapy.

**5.1.4Administration:** Cisplatin should be given immediately after preparation as a rapid intravenous injection or slow intravenous infusion.

**5.1.5Storage&Stability:** The intact vials should be stored under refrigeration. However, once reconstituted, the solution should be kept at room temperature to avoid precipitation. Due to a lack of preservatives, the solution should be used within 8 hours of reconstitution. The solution may be further diluted in a chloride containing vehicle such as D5NS, NS, or D5 1/2 NS (ppt. occurs in D5W). Cisplatin has been shown to react with aluminum needles, producing a black precipitate within 30 minutes.

### 5.2 Nedaplatin

**5.2.1**Producted by Simcere Pharmaceuticals Co., Ltd.

**5.2.2Mechanism of action:** Nedaplatin has the same ammine carrier ligands as cisplatin, but has a different leaving group, consisting of a five-membered ring structure in which glycolate is bound to the platinum ion as a bidentate ligand. Nedaplatin reacts with nucleosides to form a nucleoside-platinum complex. It has been confirmed that the types of combined bases in nedaplatin after reaction with DNA are identical to those observed in cisplatin. After uptake into cells, the glycolate portion of nedaplatin is cleaved by hydrolysis, forming “active species 1”. Active species 1 interconvert between a series of other active species, all of which exist in equilibrium. The active species binds to DNA, thereby inhibiting DNA duplication[21].

**5.2.3Pharmacokinetics:** The pharmacokinetics of nedaplatin was examined in a phase I study. Nedaplatin was given in short-term (30-min) i.v. drip infusions with the doses of 100 $mg/m^2$. Ultrafilterable platinum in plasma decreased in a biexponential mode after infusions of nedaplatin. The peak plasma concentration and AUC for free platinum were 5.31 micrograms/ml and 959 micrograms/min per ml. More than 50% of nedaplatin was excreted in the urine within the first 480 min after its administration. Thrombocytopenia was reported[16].

**5.2.4Toxicity:** The dose-limiting toxicity of nedaplatin is myelosuppression, including leucopenia, anemia, and primarily thrombocytopenia. Leukopenia and anemia often occurred at higher doses of nedaplatin, but were milder than the thrombocytopenia. Nedaplatin-induced nephrotoxicity was

reported to be characterized by apoptosis and/or necrosis, with subsequent regeneration and cystic dilatation, not only in the proximal tubules but also in the distal tubules and the collecting duct in a histopathological examination in rats[21].

**5.2.5Administration:** Based on the results of the Phase I study of single administration of nedaplatin, the maximum tolerated dose was established as 120 mg/m$^2$. Consequently, nedaplatin was initially administered alone at doses of 100 mg/m$^2$ intravenously every four weeks. The Phase I study of five days of continuous administration of nedaplatin showed that a dose of 75.5 mg/m$^2$ would be feasible over a five-day period; however, it was concluded that continuous administration of nedaplatin would offer no advantage over single administration with a four-week interval, because the area under the curve (AUC) values for free platinum at 75.5 mg/m$^2$ every five days and 100 mg/m$^2$/day were nearly equivalent[21].

## 6.0 Observation and assessment

### 6.1 Before Treatment

All patients are under standardized management for nasopharyngeal carcinoma, and they need to perform a series of examinations as well as provide relevant information to confirm pathologic diagnosis and clinical stage before admitted into trial:

a. Medical history review
b. Personal data collection
c. The present medications and treatment
d. Body examinations, include height, weight and vital signs
e. Physical examination of head and neck region, include nasopharynx and cervical lymph nodes
f. Physical examination of the nervous system
g. Nasal endoscopy and lesion biopsy
h. Blood routine
i. Urine routine
j. Blood biochemistry
k. EBV serologic tests (EBV antibodies)
l. EBV DNA was optional, depending on the laboratory availability of the participating centers.
m. EKG
n. Imaging test of tumor (enhanced MR or enhanced CT of the head and neck (CT was indicated only in patients with contraindication to MRI))
o. Chest film or CT
p. ECT bone scan
q. Abdominal ultrasonography or CT
r. PET/CT was optional and was performed at the discretion of the attending physician
s. Signed informed consent

### 6.2 During Treatment

The following aspects need to be assessed from the start of treatment to end.

a. MR of tumor should be performed before and after treatment, and CR, PR, SD or PD is evaluated with the criteria of RECIST version 1.1. Chest film and abdominal ultrasonography are reexamined after treatment.
b. The use of concomitant drugs
c. General conditions
d. Acute and late toxicities assessment (NCI-CTC, version 3.0), include hematological toxicity, gastrointestinal reactions, hepatotoxicity and nephrotoxicity, mucositis, neurotoxicity and ototoxicity, etc.
e. Peripheral neuropathy
f. Laboratory tests: blood routine and blood biochemistry are required on Day 0, Day 8, Day 15 of chemotherapy for each cycle.
g. Indirect nasopharyngoscopy is performed to examine the tumor in nasopharynx every week, and the regression of enlarged lymph nodes is observed and measured. Nasal endoscopy is performed before and after treatment course, and is also required after each cycle of chemotherapy.
h. EORTC QLQ-C30 and QLQ-H&N35 (V1.0) are used to assess patient’s quality of life, and the change of their quality of life is recorded and analyzed by week from before the beginning of

treatment to end (Week 1-6), including several important points of time such as each cycle of chemotherapy.

## 7.0 Follow up and record of events

After completion of treatment, patients were followed up at least every 3 months during the first 3 years and every 6 months thereafter until death. The nasopharynx should be assessed by endoscopy approximately 4 weeks after completion of RT. Further investigations with MRI or CT should be arranged 3 months after completion of RT. Treatment responses are also evaluated according to the RECIST. If residual disease is found, whether to treat and which treatment modalities to be employed will be decided by individual clinician. For statistical purpose, any residual disease found 16 weeks after completion of RT will be regarded as local failure. Similarly, any residual nodal disease at 16 weeks after RT is regarded as regional failure.

Follow-up procedure includes physical examination of nasopharynx and head-neck region, and plasma EBV DNA (EBV DNA was optional, depending on the laboratory availability of the participating centers) for every 3 months; abdominal sonography, chest film were routinely performed annually; enhanced MR or CT of head and neck for 3 months after radiotherapy and every one year thereafter.

Failure should preferably be confirmed by fine needle aspiration (FNA) or biopsy. In case of doubt about the origin of metastases, plasma EBV DNA and/or in situ hybridization for Epstein-Barr virus-encoded RNA (EBERs) expression in biopsy tissue should be considered for confirming nasopharynx origin. Clinical diagnosis is accepted for sites not easily accessible if classical changes are shown on imaging. The dates of diagnosis of local, nodal, and distant failure should be recorded.

The earliest date of detecting symptomatic late toxicities and the eventual maximum grade by the Late Radiation Morbidity Scoring Criteria of the Radiation Therapy Oncology Group (RTOG) and European Organization for Research and Treatment of Cancer (EORTC) should be recorded (Appendix III)

All patients will be follow-up until death and cause of death recorded. Deaths due to unknown cause are counted as death due to NPC if disease is still present at last assessment.

## 8.0 Security Measures and Quality Control

a. Provide systematic learning program for every member in the research group. Arrange one doctor in each center to take charge of tumor staging, which must be in accordance with the 7th AJCC edition, and to make sure that every patient enrolled is eligible. Patients are given random numbers to determine which group they are in.
b. Make monitoring plan of adverse effects and emergency plan.
c. Research plan is made by all participating centers and approved by Ethics Committee.
d. Develop all kinds of Standard Operation Procedures related to this study.
e. Establish standardized evaluation system to unify diagnostic criteria, curative effect judging criteria, etc.
f. Establish professional statistical plan.
g. Research staffs are trained before the study.
h. Ensure that every participating center conducts the study at the same pace.
i. Arrange quality controller, make quality control plan and check regularly.
j. Set up coordination committee, curative effect judging group and follow-up team.

## 9.0 Statistical Analysis

## 9.1 Endpoint Definitions

### 9.1.1 Primary endpoint

**Progression-free survival (PFS):** The PFS was defined as the time from random assignment to documented local or regional relapse, distant metastasis, or death from any cause, whichever occurred first.

### 9.1.2 Secondary endpoints

**9.1.2.1 Overall survival (OS)**: The OS was defined as the time from random assignment to

death from any cause or censored at the date of last follow-up.

**9.1.2.2 Locoregional relapse-free survival (LRRFS):** The LRRFS was defined as the time from random assignment to local or regional relapse, or death without experiencing the failure.

**9.1.2.3 Distant metastasis-free survival:** The DMFS was defined as the time from random assignment to distant metastasis, or death without experiencing the failure.

**9.1.2.4 Short-term response**: Nasopharyngeal tumor and cervical lymph node response will be observed and evaluated by physical examination, nasopharyngoscopy, and MRI/CT imaging 3 months later after treatment. Tumor response was classified according to RECIST, version 1.1. Complete response (CR): Disappearance of all target lesions. Any pathological lymph nodes (whether target or non-target) must have reduction in short axis to <10 mm. Partial response (PR): At least a 30% decrease in the sum of diameters of target lesions, taking as reference the baseline sum diameters. Progressive disease (PD): At least a 20% increase in the sum of diameters of target lesions, taking as reference the smallest sum on study (this includes the baseline sum if that is the smallest on study). In addition to the relative increase of 20%, the sum must also demonstrate an absolute increase of at least 5 mm. (Note: the appearance of one or more new lesions is also considered progression). Stable disease (SD): Neither sufficient shrinkage to qualify for PR nor sufficient increase to qualify for PD, taking as reference the smallest sum diameters while on study.

**9.1.2.5 Safety indicators:** Acute toxicities are assessed according to NCI-CTC version 3.0. Acute toxicities include hematological toxicity, mucositis, allergic reactions and other adverse events and serious adverse events. Late radiation toxicities were assessed using the Radiation Therapy Oncology Group and European Organization for Research and Treatment of Cancer late radiation morbidity scoring scheme. Late toxicities include neurotoxicity, ototoxicity and other complications and sequelae.

**9.1.2.6 Quality of Life**: EORTC QLQ-C30 and QLQ-H&N35 (V1.0) are used to assess life quality of patients, and the change of their life quality is recorded and evaluated weekly (Week1-6) from before the beginning of treatment to end.

**9.1.2.7 Cost-effectiveness analysis**: Cost is estimated giving consideration of direct cost such as drug fees, examination fees, ward fees and nursing fees, etc. Make cost-effectiveness analysis by calculating cost-effectiveness ratio (ratio between the direct cost and short-/long-term curative effect) and incremental cost-effectiveness ratio (ratio between increased cost and increased short-/long-term curative effect).

### 9.2 Sample Size Estimate

The primary endpoint is 2-year progression-free survival. Based on Chen's study (Chen L, Hu CS… Lancet Oncology, 2012; 13:163-171), we suppose that the 2-year progression-free survivals, about 84%, are the same between nedaplatin- and cisplatin-based chemotherapy plus IMRT in treatment of locally advanced NPC. We specify a non-inferiority margin of 10%, one-tailed α of 0.025, power of 0.85, and calculate the sample size. An estimated 169 patients are required for each group. On the assumption of 10% dropout rate, at least 372 patients (186 per group) are to be recruited

### 9.3 Stratification /Randomization Scheme

#### 9.3.1 Stratification:

Patients will be stratified according to the treatment centers (SYSCC vs. GMU vs. MZCH vs. GPU) and stage (II or III or IV).

#### 9.3.2 Randomization

Eligible patients will be randomized using a 1:1 allocation of patients to cisplatin arm and nedaplatin arm. Stratified randomization is performed within each stratum based on treatment center and clinical stages, and is also blocked. The randomized block design is conducted by GCP center in Sun Yat-sen University Cancer Center and block size will be chosen by the statistician (Prof. Qin Liu) so that each block contains the patients in equal proportion. This procedure helps to ensure both randomness and investigator blinding (the block sizes are known only to the statistician), as recommended by Friedman et al (Friedman J, Furberg, C, DeMets D. Fundamentals of clinical trials. New York: Springer-Verlag; 1998). Randomization will be generated by the statistician in opaque, sealed envelopes, labeled by stratum, which will only be unsealed after patient registration. Patients will be identified by a unique subject number that will remain constant for the duration of the study.

### 9.4 Data Management

All information about enrolled patients after registration will be sent to Sun Yat-sen University

Cancer Center for management. We have stewards taking charge of database management, and our data platform allows simultaneous input and double check.

### 9.5 Case Report Form (CRF)

The case report form is designed before the study. The CRF is required to record detailed medical history, treatment and follow-up information, and it should be easy to fill in as well as save in database.

### 9.6 Analytical Approach

The results of our study are analyzed by the intention-to treat (ITT) approach, and all eligible patients are analyzed according to the randomization scheme, including the patients whose treatment plan is changed from nedaplatin to cisplatin due to allergic reactions. Per-protocol (PP) analysis is also used to include only those who received at least one cycle of concurrent nedaplatin or cisplatin as scheduled. 95% *CI* is calculated. The Kaplan–Meier estimator is used to estimate the survival function from lifetime data, and log-rank test to compare the difference of survivals between two groups. Response rates, incidence of toxicities are compared by the chi-square test. Quality of life was analysed using a mixed effect model. Multiple prognostic factors are analyzed by Cox regression. The statistical test for PFS was one sided, and a P value < 0.025 was considered statistically significant; the left statistical tests were two-sided, and a P value < 0.05 was considered statistically significant.

**Analysis includes:**

- General information:
  The distribution and equilibrium of general factors, such as age, gender, stages, are assessed.
- Adverse effects:
  Acute and late toxicities, sequelae and complications in each arm are assessed according to NCI-CTC version 3.0.
- Short-term effect:
  CR, PR, SD or PD is evaluated with the criteria of RECIST.
- Long-term curative effect:
  2-year, 3-year, 5-year PFS, OS, LRRFS and DMFS rates are calculated according to follow-up visits.
- Analysis in each participating center:
  Data analysis is conducted in each participating center.
- Total data analysis:
  An overall analysis is conducted after data summarization.
- Stratified analysis:
  Stratified analysis is conducted according to stages.
- Life quality:
  EORTC QLQ-C30 and QLQ-H&N35 (V1.0) are used to assess life quality of patients.
- Cost-effectiveness analysis:
  Cost-effectiveness ratio and incremental cost-effectiveness ratio are calculated.

## 10.0 Ethical Considerations

**10.1**This study must be approved by an appropriate institutional ethic committee.

**10.2** An informed consent must be obtained from individual patients. Copy of the Consent Form, contact number of investigator and ethics committee will be available to patient on request.

**10.3**All serious and unexpected adverse experience or death related to the drugs or radiotherapy must be reported to the study coordinator immediately. Serious adverse events (SAE) to be reported include all deaths during or within 30 days of protocol treatment regardless of cause, grade 5 toxicity, life-threatening grade 4 toxicity, and/or unexpected toxicity. The Study Coordinator of respective center should complete form and fax this within 24 hours to the Principal Investigator (Dr. Haiqiang Mai, Tel: 020-87343643, Fax: 020-87343392), the center of clinical trials, the institutional ethic committee and Sun Yat-sen University Cancer Center. Together with the Principal Investigator, appropriate and prompt action will be taken if warranted. Reactions and deaths beyond 30 days from protocol treatment that are judged definitely unrelated to treatment should not be reported.

## 11.0 Schedule

| Time | Events |
|---|---|
| Sep, 2011----Dec, 2011 | Trial protocol development<br>Data platform construction & systematic training in research team<br>Trial initial meeting |
| Jan, 2012----Dec, 2014 | Recruitment, Assessment<br>Follow-up and detection of failure or late toxicity<br>Database completed |
| Jan, 2015----Dec, 2017 | Follow-up<br>Write research paper and get published on SCI journals (concerning 2-year PFS, response rate, adverse effects, compliance, life quality, etc.) |
| Jan, 2018----Dec, 2019 | Follow-up<br>Write paper and get published on SCI journals (concerning long-term survival and late toxicities) |

## 12.0BIBLIOGRAPHY


1. Ma J, Mai HQ, Hong MH, et al. Is the 1997 AJCC staging system for nasopharyngeal carcinoma prognostically useful for Chinese patient populations? Int J Radiat Oncol Biol Phys. 2001, 50(5):1181-1189.
2. Al-Sarraf M, LeBlanc M, Giri PG, et al. Chemoradiotherapy versus radiotherapy in patients with advanced nasopharyngeal cancer: phase III randomized Intergroup study 0099. J Clin Oncol, 1998, 16(4）:1310-1317.
3. Chan AT, Teo PM, Ngan RK, et al. Concurrent chemotherapy-radiotherapy compared with radiotherapy alone in locoregionally advanced nasopharyngeal carcinoma: progression-free survival analysis of a phase III randomized trial. J Clin Oncol, 2002, 20(8）:2038-2044.
4. Lin JC, Jan JS, Hsu CY, Liang WM, Jiang RS, Wang WY. Phase III study of concurrent chemoradiotherapy versus radiotherapy alone for advanced nasopharyngeal carcinoma: positive effect on overall and progression-free survival. J Clin Oncol, 2003, 21(4）: 631-637.
5. Zhang L, Zhao C，Peng PJ, et al. Phase III study comparing standard radiotherapy with or without weekly oxaliplatin in treatment of locoregionally advanced nasopharyngeal carcinoma： preliminary results. J Clin Oncol, 2005, 23(33)：8461-8468.
6. Wee J, Tan EH, Tai BC, et al. Randomized trial of radiotherapy versus concurrent chemoradiotherapy followed by adjuvant chemotherapy in patients with American Joint Committee on Cancer/International Union against cancer stage III and IV nasopharyngeal cancer of the endemic variety. J Clin Oncol, 2005, 23(27)：6730-6738.
7. Lee AW, Lau WH, Tung SY, et al. Hong Kong Nasopharyngeal Cancer Study Group. Preliminary results of a randomized study on therapeutic gain by concurrent chemotherapy for regionally-advanced nasopharyngeal carcinoma: NPC-9901 Trial by the Hong Kong Nasopharyngeal Cancer Study Group. J Clin Oncol, 2005, 23(28)：6966-6975.
8. Chen QY, Wen YF, Guo L, et al. Concurrent Chemoradiotherapyvs Radiotherapy Alone in Stage II Nasopharyngeal Carcinoma: Phase III Randomized Trial, Journal of the National Cancer Institute: 2011; 103(23): 1761-1770.
9. Huncharek M, Kupelnick B. Combined chemoradiation versus radiation therapy alone in locally advanced nasopharyngeal carcinoma: results of a meta-analysis of 1,528 patients from six randomized trials. Am J Clin Oncol. 2002, 25(3): 219-223.
10. Langendijk JA, Leemans CR, Buter J, Berkhof J, Slotman BJ. The additional value of chemotherapy to radiotherapy in locally advanced nasopharyngeal carcinoma: a meta-analysis of the published literature. J Clin Oncol. 2004, 22(22): 4604-4612.
11. Baujat B, Audry H, Bourhis J, et al. Chemotherapy in locally advanced nasopharyngeal carcinoma: an individual patient data meta-analysis of eight randomized trials and 1753 patients. Int J Radiat

Oncol Biol Phys. 2006, 64(1): 47-56.
12. Yau TK, Lee AW, Wong DH, et al. Treatment of Stage IV(A-B) nasopharyngeal carcinoma by induction-concurrent chemoradiotherapy and accelerated fractionation: impact of chemotherapy schemes. Int J Radiat Oncol Bio Phys. 2006, 66 (4): 1004-10.
13. Chitapanarux I, Lorvidhaya V, Kamnerdsupaphon P, et al. Chemoradiation comparing cisplatin versus carboplatin in locally advanced nasopharyngeal cancer: randomised, non-inferiority, open trial. Eur J cancer. 2007 43 (9): 1399-406.
14. Huang PY, Mai HQ, Hong MH, et al. Induction-concurrent chemoradiotherapy versus induction chemotherapy and radiotherapy for locoregionally advanced nasopharyngeal carcinoma. Chin J Cancer, 2009, 28(10): 1033-1042.
15. Tanaka T, Yukawa K, Umesaki N. Radiation reduces carboplatin sensitivity and enhances nedaplatin sensitivity in cervical squamous cell carcinoma in vitro. Eur J Gynaecol Oncol. 2007, 28(5): 352-5.
16. Sasaki Y, Tamura T, Eguchi K, et al. Pharmacokinetics of (glycolate-0,0')-diammine platinum (II), a new platinum derivative, in comparison with cisplatin and carboplatin. Cancer Chemother Pharmacol. 1989, 23(4): 243-246.
17. Desoize B, Madoulet C. Particular aspects of platinum compounds used at present in cancer treatment. Crit Rev Oncol Hematol.2002 42(3): 317-325.
18. Fujii M, Tokumaru Y, Imanishi Y, Kanke M, Kanzaki J, Ohno Y. Combination chemotherapy with nedaplatin and 5-FU for head and neck cancer. Gan To Kagaku Ryoho. 1998; 25(1): 53-58.
19. Fuwa N, Kodaira T, Tachibana H, Nakamura T, Daimon T. Dose escalation study of nedaplatin with 5-fluorouracil in combination with alternating radiotherapy in patients with head and neck cancer. Jpn J Clin Oncol. 2007, 37(3): 161-167.
20. Zheng J, Wang G, Yang GY, et al. Induction chemotherapy with nedaplatin with 5-FU followed by intensity-modulated radiotherapy concurrent with chemotherapy for locoregionally advanced nasopharyngeal carcinoma. Jpn J Clin Oncol. 2010, 40(5): 425-431.
21. Desoize B, Madoulet C. Particular aspects of platinum compounds used at present in cancer treatment. Crit Rev Oncol Hematol. 2002:42(3): 317-25.

**Appendix I**

**STAGING CRITERIA – the 7th AJCC edition**[2]

Nasopharynx (T)

| | |
|---|---|
| T1 | Nasopharynx, soft tissue of oropharynx and/or nasal fossa without parapharyngeal extension |
| T2 | Parapharyngeal extension |
| T3 | Invades bony structures and/or paranasal sinuses |
| T4 | Intracranial extension, involvement of cranial nerves, infratemporal fossa, hypopharynx, orbit |

Regional Lymph Node (N)

| | |
|---|---|
| N1 | Unilateral lymph node(s) < 6 cm, above supraclavicular fossa, and/or unilateral or bilateral,retropharyngeal lymph node(s) < 6 cm |
| N2 | Bilateral lymph node(s) < 6 cm, above supraclavicular fossa |
| N3 | (a) >6 cm or<br>(b) in the supraclavicular fossa |

Distant Metastasis (M)

| | |
|---|---|
| M0 | No distant metastasis |
| M1 | Distant metastasis |

Stage Grouping

| | | | |
|---|---|---|---|
| Stage 0 | Tis | N0 | M0 |
| Stage I | T1 | N0 | M0 |
| Stage II | T1 | N1 | M0 |
| | T2 | N0 | M0 |
| | T2 | N1 | M0 |
| Stage III | T3 | N0, N1 | M0 |

| | | | |
|---|---|---|---|
| | T1, T2, T3 | N2 | M0 |
| Stage IVA | T4 | N0, N1, N2 | M0 |
| Stage IVB | Any T | N3 | M0 |
| Stage IVC | Any T | Any N | M1 |

**Appendix II**

**Performance Status (Karnofsky scale)**

| | |
|---|---|
| 100 | No complaints; No evidence of disease |
| 90 | Able to carry on normal activity; minor signs or symptoms of disease |
| 80 | Able to carry on normal activity with effort; Some signs or symptoms of disease |
| 70 | Cares for self; unable to carry on normal activity or to do active work |
| 60 | Requires occasional assistance but is able to care for most personal needs |
| 50 | Requires considerablel assistance and frequent medical care |
| 40 | Disabled; requires special care and assistance |
| 30 | Severely disabled; hospitalization indicated, although death not imminent |
| 20 | Very sick; hospitalization necessary; requires active supportive treatment |
| 10 | Moribund; fatal processes progressing rapidly |
| 0 | Dead |

**Appendix III**

**Toxicity Criteria**
<u>INSTRUCTIONS</u>

1. Toxicity grade should reflect the most severe degree occurring during the evaluated period, not an average.
2. When two criteria are available for similar toxicity, the one resulting in the more severe grade should be used.
3. Toxicity grade = 5 if that toxicity caused the death of the patient.
4. Refer to detailed toxicity guidelines in CTCAE system for acute induction chemotherapy and chemoradiotherapy toxicity not covered on this table.
5. Refer to detailed toxicity guidelines in RTOG system for late radiation toxicity not covered on this table, some items are modified for better evaluation.
6. The evaluator must attempt to discriminate between disease/treatment and related signs/symptoms.
7. An accurate baseline prior to start of therapy is necessary.

## Acute chemoradiotherapy toxicity (CTCAE System)

| Toxicity | Grade 1 | Grade 2 | Grade 3 | Grade 4 | Grade 5 |
|---|---|---|---|---|---|
| Rash: dermatitis associated with radiation | Faint erythema or dry desquamation | Moderate to brisk erythema; patchy moist desquamation, mostly confined to skin folds and creases; moderate edema | Moist desquamation other than skin folds and creases; bleeding induced by minor trauma or abrasion | Skin necrosis ulceration of full thickness dermis; spontaneous bleeding from involved site | Death |
| Mucositis/stomatitis(clinical exam) | Erythema of the mucosa | Patchy ulcerations or pseudomembranes | Confluent ulcerations or pseudomembranes; bleeding with minor trauma | Tissue necrosis; significant spontaneous bleeding; life-threatening consequences | Death |
| Anorexia | Loss of appetite without alteration in eating habits | Oral intake altered without significant weight loss or malnutrition; oral nutritional supplements indicated | Associated with significant weight loss or malnutrition (e.g., inadequate oral caloric and/or fluid intake); IV fluids, tube feedings or TPN indicated | Life-threatening consequences | Death |
| Nausea | Loss of appetite without alteration in eating habits | Oral intake decreased without significant weight loss, dehydration or malnutrition; IV fluids indicated <24 hrs | Inadequate oral caloric or fluid intake; IV fluids, tube feeding, or TPN indicated ≥24hrs | Life-threatening consequences | Death |
| Vomiting | 1 episode in 24 hrs | 2 - 5 episodes in 24 hrs; IV fluids indicated <24 hrs | ≥6 episodes in 24 hus; IV fluids or TPN indicated ≥24hrs | Life-threatening consequences | Death |
| Dry mouth | Symptomatic (dry or thick saliva) without significant dietary alteration; unstimulated saliva flow >0.2 ml/min | Symptomatic and significant oral intake alteration (e.g., copious water, other lubricants, diet limited to purees and/or soft, moist foods); unstimulated saliva 0.1 to 0.2 ml/min | Symptoms leading to inability to adequately aliment orally, IV fluids, tube feedings, or TPN indicated unstimulated saliva <0.1ml/min | - | - |
| Dysphagia | Symptomatic, able to eat regular diet | Symptomatic and altered eating/swallowing (e.g., altered dietary habits, oral supplements); IV fluids indicated <24 hrs | Symptomatic and severely altered eating/swallowing(e.g. inadequate oral caloric or fluid intake);IV fluids,tube feedings,or TPN indicated ≥24hrs | Life-threatening consequences(e.g. obstruction, perforation) | Death |
| Diarrhea | Increase of <4 stools per day over baseline; mild increase in ostomy output compared to baseline | Increase of 4 - 6 stools per day over baseline; IV fluids indicated <24hrs; moderate increase in ostomy output compared to baseline; not interfering with ADL | Increase of ≥7 stools per day over baseline; incontinence; IV fluids indicated ≥24hrs; hospitalization; severe increase in ostomy output compared to baseline; interfering with ADL | Life-threatening consequences (e.g., hemodynamic collapse) | Death |
| ALT | >ULN - 2.5 x ULN | >2.5 - 5.0 x ULN | >5.0–20.0×ULN | >20.0×ULN | - |
| AST | >ULN - 2.5 x ULN | >2.5 - 5.0 x ULN | >5.0–20.0×ULN | >20.0×ULN | - |
| CRE | >ULN - 1.5 x ULN | >1.5 - 3.0 x ULN | >3.0–6.0×ULN | >6.0×ULN | Death |
| Hearing (without | - | Hearing loss not requiring hearing aid or | Hearing loss requiring hearing aid or | Profound bilateral hearing loss | - |

| | | | | | |
|---|---|---|---|---|---|
| monitoring program) | | intervention (i.e., not interfering with ADL) | intervention (i.e., interfering with ADL) | (>90dB) | |
| Neuropathy: sensory | Asymptomatic; loss of deep tendon reflexes or paresthesia (including tingling) but not interfering with function | Sensory alteration or paresthesia (including tingling), interfering with function, but not interfering with ADL | Sensory alteration or paresthesia interfering with ADL | Disabling | Death |
| Neuropathy: motor | Asymptomatic, weakness on exam/testing only | Symptomatic weakness interfering with function, but not interfering with ADL | Weakness interferingwith ADL; bracing or assistance to walk (e.g., cane or walker) indicated | Life-threatening;disabling(e.g., paralysis) | Death |
| Leukocytes | 3.0×109/L-<ULN | 2.0–<3.0×109/L | 1.0–<2.0×$10^9$/L | <1.0×$10^9$/L | Death |
| Neutrophils | 1.5×109/L-<ULN | 1.0–<1.5×109/L | 0.5–<1.0×$10^9$/L | <0.5×$10^9$/L | Death |
| Hemoglobin | 100g/L-<ULN | 80–<100 g/L | 65–<80 g/L | <65 g/L | Death |
| Platelets | 75.0×109/L-<ULN | 50.0–<75.0×109/L | 25.0–<50.0×$10^9$/L | <25.0×$10^9$/L | Death |
| Weight loss | 5 to <10% from baseline; intervention not indicated | 10 - <20% from baseline; nutritional support indicated | ≥20% of baseline; tube feeding or TPN indicated | - | - |
| lethargy | Mild fatigue over baseline | Moderate or causing difficulty performing some ADL | Severe fatigue interfering with ADL | Disabling | - |
| Hair loss /alopecia (scalp or body) | Thinning or patchy | Complete | - | - | - |
| Allergic reaction | Transient flushing or rash; drug fever <38 degrees C (<100.4 degrees F) | Rash; flushing; urticaria; dyspnea; drug fever >=38 degrees C (>=100.4 degrees F) | Symptomatic bronchospasm, with or without urticaria; parenteral medication(s) indicated; allergy-ralated edema /angioedema; hypotension | Anaphylaxis | Death |
| Fever (In the absence of neutropenia, where neutropenia is defined as ANC<1.0×$10^9$/L) | 38.0 - 39.0℃ | >39.0 - 40.0℃ | >40℃,for≤24hrs | >40℃,for> 24hrs | Death |

| | | | | | |
|---|---|---|---|---|---|
| Infection(documentedclinically or microbiologically) withGrade 3 or 4 neutrophils (ANC <1.0 x $10^9$/L) | - | Localized, local intervention indicated | IV antibiotic, antifungal, orantiviral intervention indicated; interventional radiology or operative intervention indicated | Life-threateningconsequences (e.g.,septic shock,hypotension,acidosis,necrosis) | Death |
| Teeth | Surface stains; dental caries; restorable, without extractions | Less than full mouth extractions; tooth fracture or crown amputation or repair indicated | Full mouth extractions indicated | - | - |

## Late RT toxicity(RTOG/EORTC System)

| Toxicity | Grade 1 | Grade 2 | Grade 3 | Grade 4 |
|---|---|---|---|---|
| Temporal lobe necrosis | Mild headache<br>Slight lethargy | Moderate headache<br>Great lethargy | Severe headaches;<br>severe CNS dysfunction (partial loss of power or dyskinesia)<br>(major intellectual impairment;<br>persistent & minor mood/ personality change;<br>cannot perform simple task) | Seizures; paralysis;<br>Coma;Required surgical treatment (complete loss of memory; complete disorientation;totaldisintegration;incapable of self-care) |
| Spinal cord/Brainstem | Mild L'Hermitte's syndrome | Severe L'Hermitte's syndrome | Objective neurological findings<br>(partial sensory loss;<br>persistent motor weakness;<br>incomplete sphincter control) | Mono, para quadriplegia(total sensory loss;complete motor power loss;<br>complete incontinence) |
| Peripheral nerves | Mild numbness; negligible paresthesia or decrease in power | Intermittent paresthesia; 25% decrease in power | Persistent paresthesia;<br>50% decrease in power | Total sensory loss;<br>complete motor power loss |
| Adrenal | Asymptomatic, intervention not indicated | Mild drowsiness & weakness; < 50% decrease in cortisol | Severe drowsiness & weakness<br>darkened skin;<br>> 50% decrease in cortisol | Paralysis;<br>Coma |
| Ear | Negligible pain/ otitis/ tinnitus/ hearing loss | Intermittent pain/ otitis;<br>intermittent tinnitus; moderate hearing loss | Persistent pain/ otitis; persistent (daily) tinnitus;<br>severe hearing loss, frequent difficulties with loud speech | Refractory pain/ otitis; refractory (constant) tinnitus;<br>Complete deafness |

| | | | | |
|---|---|---|---|---|
| Eyeball | Asymptomatic cataract<br>Minor corneal ulceration or keratitis | Symptomatic cataract<br>Moderate corneal ulceration<br>Minor retinopathy or glaucoma | Severe keratitis;<br>severe retinopathy or detachment<br>severe glaucoma<br>(severe loss of vision but able to perform daily activity) | Panopthalmitis<br>blindness<br>(unable to perform daily activity) |
| Bone | Asymptomatic<br>No growth retardation<br>Reduced bone density | Moderate pain or tenderness<br>Growth retardation<br>Irregular bone sclerosis | Severe pain or tenderness; complete arrest of bone growth; dense bone sclerosis (regular narcotic) | Necrosis;<br>spontaneous fracture<br>(surgical tervention) |
| Trismus | Mild joint stiffness<br>Slight limitation of movement | Moderate stiffness<br>Intermittent or moderate joint pain<br>Moderate limitation of movement | Severe joint stiffness; severe pain;<br>severe limitation of movement<br>(dental gap 0.5–1 cm) | Necrosis;<br>complete fixation<br>(dental gap < 0.5 cm) |
| Dry mouth | Slight dryness of mouth<br>Good response on stimulation | Moderate dryness of mouth<br>Poor response on stimulation | Complete dry mouth, not response to any stimulus | Fibrosis |
| Subcutaneous tissue<br>Soft tissue/ muscle | Slight induration (fibrosia) and loss of subcutaneous fat | Moderate fibrosis but asymptomatic<br>Slight field contracture<br><10% linear reduction | Severe induration;severe loss of subcutaneous tissue; contracture > 10% linear reduction<br>(secondary dysfunction) | Necrosis<br>(total dysfunction) |
| Soft tissue<br>skin/ mucosa | Slight atrophy<br>Pigmentation change<br>Some hair loss | Patch atrophy; Moderate telangiectasia; Total hair loss | Marked atrophy;<br>gross telangiectasia($\geq$ 50%) | Ulceration |
