# Supplementary material for: Effect of Concurrent Chemoradiotherapy With Nedaplatin vs Cisplatin on the Long-term Outcomes of Survival and Toxic Effects Among Patients With Stage II to IVB Nasopharyngeal Carcinoma: A 5-Year Follow-up Secondary Analysis of a Randomized Clinical Trial
Source: JAMA Netw Open. 2021 Dec 20;4(12):e2138470. doi: 10.1001/jamanetworkopen.2021.38470 (PMC8689390; doi:10.1001/jamanetworkopen.2021.38470)
Supplement: Supplement 2. — eTable 1. Comparison of Survival Outcomes Between the Nedaplatin and Cisplatin Groups eTable 2. The 5-Year Survival Rate for the Intention-to-Treat and Per-Protocol Analysis eTable 3. Factors Associated With Grade 3–4 Auditory/Hearing Toxicities in the Safety Populations eFigure 1. Progression-Free Survival (A), Overall Survival (B), Cumulative Incidence of Distant Metastasis (C), and Cumulative Incidence of Locoregional Relapse (D) in the Per-Protocol Populations eFigure 2. Cumulative Incidence of Grade 3–4 Auditory/Hearing Toxicities in the Safety Populations With Regards to Different Treatment Group [file jamanetwopen-e2138470-s002.pdf]

Supplemental Online Content

Tang QN, Liu LT, Qi B, et al. Effect of concurrent chemoradiotherapy with nedaplatin vs cisplatin on the long-term outcomes of survival and toxic effects among patients with stage II to IVB nasopharyngeal carcinoma: a 5-year follow-up secondary analysis of a randomized clinical trial. *JAMA Netw Open*. 2021;4(12):e2138470. doi:10.1001/jamanetworkopen.2021.38470

eTable 1. Comparison of Survival Outcomes Between the Nedaplatin and Cisplatin Groups

eTable 2. The 5-Year Survival Rate for the Intention-to-Treat and Per-Protocol Analysis

eTable 3: Factors Associated With Grade 3–4 Auditory/Hearing Toxicities in the Safety Populations

eFigure 1: Progression-free Survival (A), Overall Survival (B), Cumulative Incidence of Distant Metastasis (C), and Cumulative Incidence of Locoregional Relapse (D) in the Per-Protocol Populations

eFigure 2: Cumulative Incidence Of Grade 3–4 Auditory/Hearing Toxicities In The Safety Populations With Regards To Different Treatment Group

This supplemental material has been provided by the authors to give readers additional information about their work.

© 2021 Tang QN et al. *JAMA Network Open*.

eTable 1: Comparison of survival outcomes between the nedaplatin and cisplatin groups

**Status**	**Incidence, No. (%)**	
	Intention–to–treat population	Per–protocol population
**Progression**		
Cisplatin group	41 (20.4)	40 (20.2)
Nedaplatin group	45 (22.4)	43 (21.7)
**Death**		
Cisplatin group	23 (11.4)	23 (11.6)
Nedaplatin group	26 (12.9)	25 (12.6)
**Distant failure**		
Cisplatin group	31 (15.4)	30 (15.2)
Nedaplatin group	21 (10.4)	19 (9.6)
**Locoregional failure**		
Cisplatin group	16 (8.0)	16 (8.1)
Nedaplatin group	24 (11.9)	23 (11.7)

Data are n (%) or rate.

© 2021 Tang QN et al. *JAMA Network Open*.

eTable 2. The 5-year survival rate for the intention-to-treat and per-protocol analysis

Endpoint	Intention-to-treat population		Per-protocol population	
	5-year survival (95% CI)	*p* value	5-year survival (95% CI)	*p* value
Progression-free survival		$P_{non\text{-}inferiority}$=0.002		$P_{non\text{-}inferiority}$=0.003
Cisplatin	81.4 (75.9−86.9)		81.1 (75.6−86.6)	
Nedaplatin	79.8 (74.1−85.5)		80.3 (74.6−86.0)	
Difference	1.6 (-6.3−9.5)		0.8 (-7.1−8.7)	
Overall survival		$P_{log\text{-}rank}$=0.630		$P_{log\text{-}rank}$=0.723
Cisplatin	89.4 (85.1−93.7)		89.2 (84.9−93.5)	
Nedaplatin	88.8 (84.3−93.3)		89.0 (84.5−93.5)	
Difference	0.6 (-5.6−6.8)		0.2 (–6.0−6.4)	

CI, confidence interval

© 2021 Tang QN et al. *JAMA Network Open*.

eTable 3: Factors associated with grade 3–4 auditory/hearing toxicities in the safety populations.

Factors	OR (95%CI)	P value
**Sex**		
Men	reference	
Women	1.44 (0.75-2.77)	0.280
**Age**		
<45	reference	
≥45	1.53 (0.85-2.75)	0.155
**Histology**		
WHO II	reference	
WHO III	0.45 (0.08-2.44)	0.354
**Karnofsky sore**		
70-80	reference	
90-100	0.28(0.04-2.15)	0.220
**Stage**		0.333
II	reference	
III	0.91 (0.37-2.25)	0.845
IVA	1.45 (0.51-4.13)	0.486
IVB	0.24 (0.03-2.15)	0.204
**Treatment group**		
Cisplatin	reference	
Nadaplatin	0.51 (0.28-0.93)	0.027

© 2021 Tang QN et al. *JAMA Network Open*.

eFigure 1: Progression-free survival (A), overall survival (B), cumulative incidence of distant metastasis (C), and cumulative incidence of locoregional relapse (D) in the per-protocol populations.

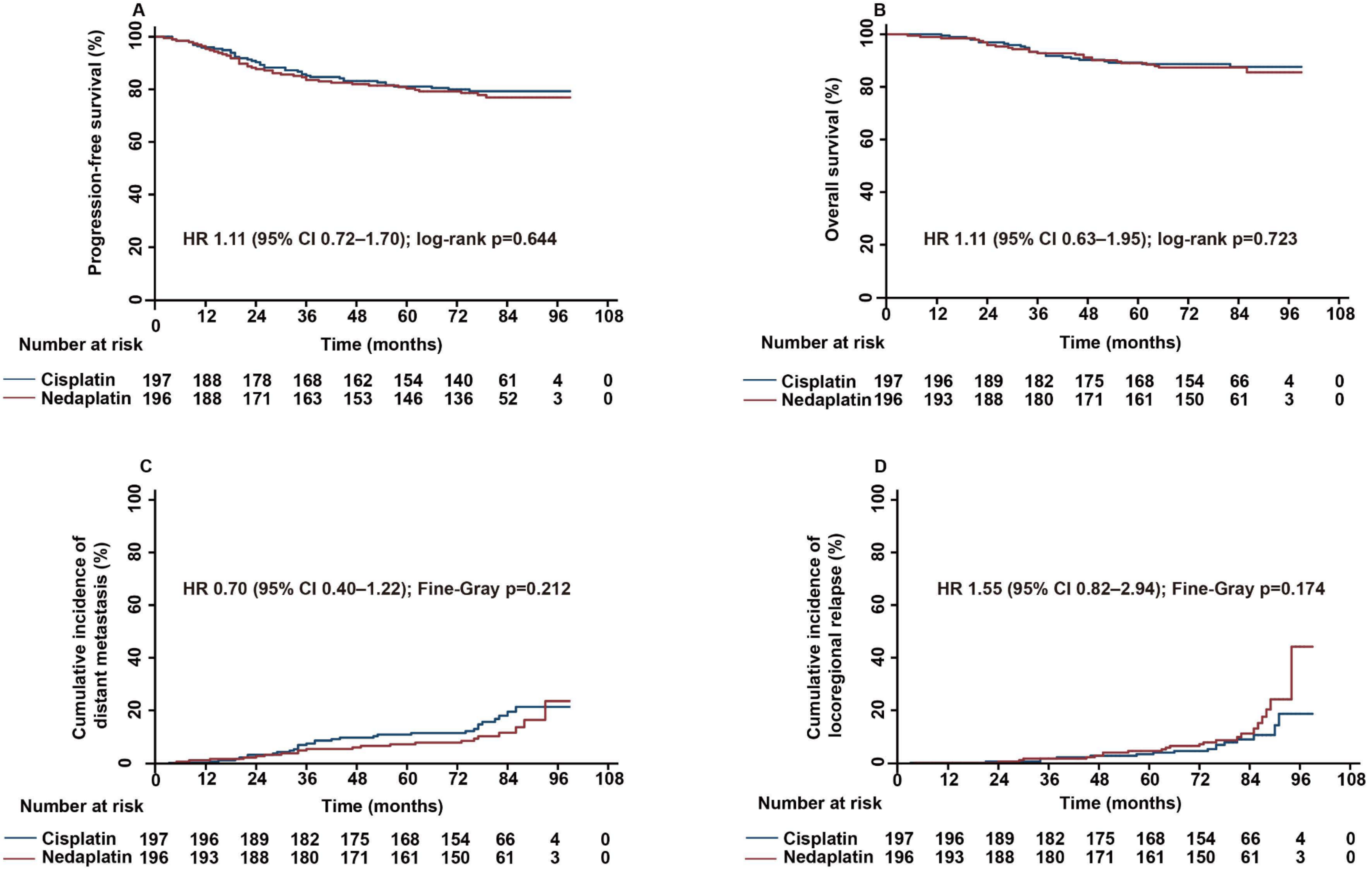

© 2021 Tang QN et al. *JAMA Network Open*.

eFigure 2: Cumulative incidence of grade 3–4 auditory/hearing toxicities in the safety populations with regards to different treatment group.

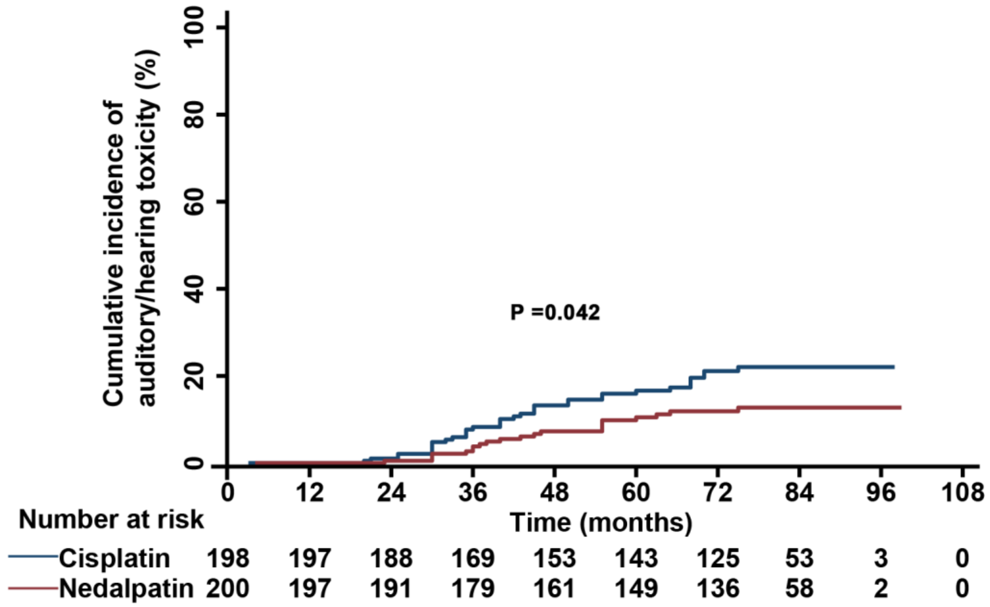

© 2021 Tang QN et al. *JAMA Network Open*.
