# Supplementary material for: Effect of Concurrent Chemoradiotherapy With Nedaplatin vs Cisplatin on the Long-term Outcomes of Survival and Toxic Effects Among Patients With Stage II to IVB Nasopharyngeal Carcinoma: A 5-Year Follow-up Secondary Analysis of a Randomized Clinical Trial
Source: JAMA Netw Open. 2021 Dec 20;4(12):e2138470. doi: 10.1001/jamanetworkopen.2021.38470 (PMC8689390; doi:10.1001/jamanetworkopen.2021.38470)
Supplement: Supplement 3. — Data Sharing Statement [file jamanetwopen-e2138470-s003.pdf]

Data Sharing Statement

Tang. Effect of Concurrent Chemoradiotherapy With Nedaplatin vs Cisplatin on the Long-term Outcomes of Survival and Toxic Effects Among Patients With Stage II to IVB Nasopharyngeal Carcinoma. *JAMA Netw Open*. Published December 20, 2021.
doi:10.1001/jamanetworkopen.2021.38470

Data
Data available: No
